# What is the prevalence of musculoskeletal problems in the elderly population in developed countries? A systematic critical literature review

**DOI:** 10.1186/2045-709X-20-31

**Published:** 2012-09-24

**Authors:** René Fejer, Alexander Ruhe

**Affiliations:** 1 The Research Department, the Spine Centre of Southern Denmark, Hospital Lillebaelt, Middelfart, Denmark; 2 Private practice, Praxis fuer Chiropraktik Wolfsburg, Wolfsburg, Germany

**Keywords:** Systematic literature review, Musculoskeletal disease, Elderly population, Osteoarthritis, Rheumatoid arthritis, Osteoporosis, Back pain

## Abstract

**Background:**

The proportion of older people will be tripled by the year 2050. In addition, the incidence of chronic musculoskeletal (MSK) conditions will also increase among the elderly people. Thus, in order to prepare for future health care demands, the magnitude and impact of MSK conditions from this growing population is needed. The objective of this literature review is to determine the current prevalence of MSK disorders in the elderly population.

**Methods:**

A systematic literature search was conducted in Pubmed on articles in English, published between January 2000 and July 2011. Studies from developed countries with prevalence estimates on elderly people (60+) on the following MSK conditions were included: Non-specific extremity pain, rheumatoid arthritis, osteoarthritis, osteoporosis, and back pain. The included articles were extracted for information and assessed for risk of bias.

**Results:**

A total of 85 articles were included with 173 different prevalence estimates. Musculoskeletal disorders are common in the elderly population, but due to heterogeneity of the studies, no general estimate on the prevalence of MSK can be determined. Women report more often MSK pain than men. Overall, prevalence estimates either remain fairly constant or increase slightly with increasing age, but with a tendency to decrease in the oldest (80+) people.

**Conclusions:**

Musculoskeletal disorders remain prevalent in the elderly population. Given the increasing proportion of elderly population in the world population and the burden of MSK diseases among the elderly people, efforts must be made to maintain their functional capacity for as long as possible through optimal primary and secondary health care.

## Background

According to the United Nations (UN), the proportion of older people (i.e. aged 60 and over) will triple over the next 40 years and will account for more than 20% of the world’s population by year 2050 [1]. In addition, it is estimated that one in five of the elderly population will be more than 80 years old in 2050. The exponential increase of elderly people is mainly due to a rise in life expectancy, especially in the developing countries. Along with the rise in the life expectancy there is also a rise in the incidence of non-communicable chronic conditions which again leads to increasing morbidity and disability [2]. According to the World Health Organization (WHO), one of the major disabling conditions among the elderly population is musculoskeletal (MSK) disorders [3,4]. The WHO has specifically identified four major disabling MSK conditions: osteoarthritis (OA), rheumatoid arthritis (RA), osteoporosis (OP), and back pain (BP) [4].

In 1998, the Bone and Joint Decade (BJD) 2000–2010 collaboration was initiated and endorsed by the UN and WHO, with the overall goal to reduce the burden and cost of MSK diseases [5,6]. In 2003, the WHO’s Global Burden of Disease study and the Bone and Joint Monitoring Project conducted a large report on the burden of MSK disorders through the existing data on the four major MSK conditions (OA, RA, OP, and low back pain (LBP)) [4,5]. From this report, it is clear that the burden of these major MSK conditions increases with age.

From a health care perspective, the rising proportion and burden of older people demands that health care professionals increase their awareness of the health and disability of this particular population. Accordingly, there is a need to better understand the current magnitude and impact of MSK conditions from this growing population.

The aim of this paper is to estimate the current prevalence of musculoskeletal disorders in the elderly population by conducting a systematic literature review. Specifically, the objective was to estimate the prevalence of non-specific musculoskeletal pain, OA, RA, OP, and BP among older people in developed countries. Any methodological shortcomings will be discussed and future recommendations will be provided.

## Methods

### Definitions

Musculoskeletal pain in this review refers to the following five overall conditions: 1) non-specific MSK pain in the extremities, 2) RA, 3) OA, 4) OP (either spine or hip or a combination of both), and 5) BP (i.e. neck pain (NP), mid back pain (MBP), and LBP). The older population is defined as people aged 60 and over according to the UN’s cut-off criterion [1]. The term “magnitude” in this review refers to the relative size (i.e. prevalence) of the selected MSK conditions. Hence, the quality of life, cost-of-illness, or social/personal burden of MSK disorders is not included. Developed countries are defined as countries with an advanced economy according to the International Monetary Fund, which includes 35 countries (Additional file 1) [7].

### Search design

A systematic literature search was conducted in Pubmed (http://www.pubmed.org) and included studies published between January 1^st^ 2000 and July 1^st^ 2011. The time-period was chosen in order to only include studies published after the WHO reports [3,4]. Search terms included both free text and MeSH terms and were combined by Boolean terms (AND, OR, NOT) (Additional file 2). The following main terms were included: “musculoskeletal”, “rheumatoid arthritis”, “osteoarthritis”, and “osteoporosis”. The MeSH terms were limited to only include studies containing “epidemiology”, “etiology”, or “diagnosis”. These were again combined with “prevalence”, “cross-sectional studies”. The search was limited by type of papers (review, government publications, technical reports or journal articles), age (MeSH terms: “aged” and “aged, 60 and over”) and finally restricted to English language only. No additional search was conducted. The retrieval of potentially relevant articles was conducted in two phases by one examiner. The first phase focused on identifying relevant studies through the title and abstract. This was followed by retrieval of all full-text articles for further eligibility. As Pubmed adds papers or change MeSH terms retrospectively, the search was repeated after July 1^st^. The last search was conducted September 1^st^ 2011. No additional searches were conducted, nor were any authors contacted.

### Eligibility criteria

Only observational studies from developed countries that reported specific MSK disorders on older people aged 60 and over were included. Thus, studies reporting general MSK pain were excluded. Preferably, the study sample had to represent the general population, but as some individuals may live in nursing homes etc., such studies were also accepted. Table 1 lists the full inclusion and exclusion criteria used in this literature review.

**Table 1:** Inclusion and exclusion criteria

**Inclusion**	**Exclusion**
· Original observational studies or reports; primarily cross-sectional and cohort studies	· If more than one article presenting results from the same study existed then only the most relevant article was included.
· Studies reporting results specifically on people aged 60 and over	· No reviews, experimental or clinical trials, or studies with subsample of the original study sample, unless it is still a representative sample and reports new relevant information
· Representative of the general population (study samples from nursing homes, etc. are accepted)	· No working populations
· Only following musculoskeletal (MSK) conditions: 1)Non-specific extremity MSK 2)Back pain (+ divided by region) 3)Osteoarthritis in larger joints of the extremities (i.e. shoulder, elbow, hand/wrist, hip, knee, ankle/foot) 4) Rheumatoid arthritis 5) Osteoporosis	· No native/aboriginal populations
· Studies from developed countries only (e.g. countries with “advanced economies” according to IMF)	· Studies reporting general MSK pain with no specific anatomical area
· Any type of prevalence/incidence	· No traumatic related injuries
· Prevalence/incidence estimates specifically on people aged 60 and over	· No secondary MSK conditions (i.e. osteoporotic fractures)
· In studies with results from more than one period/survey, only the latest	· No combined anatomical sites (e.g. neck + shoulder pain), except for back pain which is usually low back pain.
· year was included	· No OA in minor joints (such as in a single phalanx joint, facet joints, etc.)
	· Indirect/weighted/adjusted prevalence estimates.

### Extraction of information

All core information from the included studies was extracted by an unblinded examiner. The most relevant information were: Article details, study objective(s), study design, method of data collection, sampling method and sample data, disease definition, and outcome data (Table 2). If the included study referred to another reference (i.e. another paper, report, or website) for a more detailed description of the study cohort, then that reference was perused for additional information if it was accessible.

**Table 2:** List of items extracted from each article

	
1.	Article details (author(s), title, country, source)
2.	Objective(s) of study
3.	Study design (cross-sectional or cohort/longitudinal)
4.	Method of data collection (registry, questionnaire, interview, examination, etc.)
5.	Sampling method and sample data (age, gender ratio, target population, study sample, response rate)
6.	Description of MSK condition (definition, type and validation of questionnaire)
7.	Outcome data (type of prevalence/incidence, results (including gender and age estimates, 95% CI)
8.	Own remarks or conclusion

### Risk of bias assessment

The quality of each study was determined by assessing the risk of bias [8]. Recently, Viswanathan et al. have identified 29 practical and validated items that may be used to evaluate the risk of bias and precision of observational studies [9]. This bank of items covers a range of different study designs and the authors have provided instructions as to what items to use depending on the studies under assessment. Thus, only items related to our main objectives were identified and criteria for each item were defined to fit our main objective (Table 3). The layout of the questionnaire was slightly modified for practical reasons, but no other changes were made. The chosen items focused on selection bias, information bias, and the overall interpretation of each study. Relevant criteria to assist in determining the risk of bias in a study were specified to each item. No validation of the included items was performed.

**Table 3:** Items chosen to assess risk of bias of the included studies

Item number from original study*	**Dimension of bias**	**Methods domain**	**Assessment question**	**Criteria / definitions / categories**
Q2	Selection bias	Sample definition and selection	· Are critical inclusion/exclusion criteria clearly stated?	· Target population described?
				· Ascertainment procedure for target sample described?
				· Study sample representative of the target population described?
				· Age range, gender, etc. described?
				· Specific inclusion/exclusion criteria stated?
				· Sample size described?
Q3	Information bias	Sample definition and selection	· Are the inclusion/exclusion criteria measured using valid and reliable measures	· Ascertainment procedure: Random, stratified, cluster, etc. (*if applicable*)
				· Registry (census, GP databases) (reporting bias?) (*if applicable*)
				· Medical records (clinical or hospital records) (*if applicable*)
				· Non-response analysis (non-response bias) (*if applicable*)
				· Sample size: is it justified or is a power calculation provided?
Q14	Information bias	Soundness of information	· Are outcome measures assessed using valid and reliable measures?	· Questionnaire (is it valid and/or reproducible?) (*if applicable*)
				· Registry (i.e. census, GP databases) (reporting bias?) (*if applicable*)
				· Interviewing bias (i.e. structured, semi-structured, objective) (*if applicable*)
				· Self-reporting (risk of recall bias; shorter recall better than longer recall) (*if applicable*)
				· Observation, examination procedure (observer bias?) (*if applicable*)
· Q7	· Performance bias	· Exposure	· What is the level of detail in describing the outcome?	· Definition of the MSK condition; anatomical, physiological. (*required*)
				· Definition of symptom(s) (pain, problem, other) (*required*)
				· Definition of period of symptom(s) (*required, only if applicable*)
				· Description of pain intensity (*if applicable, not required*)
			· Overall judgment	· Low risk of bias: Bias, if present, is unlikely to alter the results seriously
				· Unclear risk of bias: Impossible to determine risk of bias (either missing or not described well enough)
				· High risk of bias: Bias may alter the results seriously

* Viswanathan M, Berkman ND. Development of the RTI item bank on risk of bias and precision of observational studies. J Clin Epidemiol 2011, 65:163-178.

### Data analysis

The extracted data was presented in separate tables for each of the included MSK conditions. In studies where the results were only presented graphically, best effort was made to determine the prevalence estimates from the graphs (without decimals). Both total and gender prevalence estimates as well as age related changes were reported when possible. In addition, the attempt was made to present pooled means of prevalence estimates on fairly homogeneous studies.

## Results

### Search results

**Figure 1:**
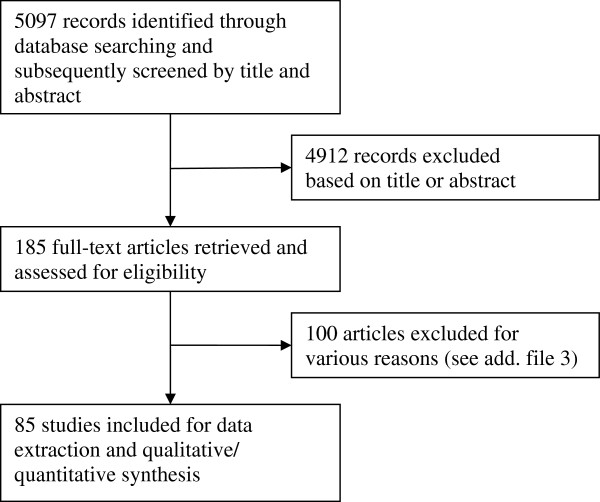
Flow chart of search results.

In total, 5097 articles were found through the search strategy (Figure 1). Based on either their title or abstract, 185 were subsequently retrieved and reviewed. Of these, 100 articles were rejected, mainly because prevalence estimates on elderly aged 60 and over was not reported or could not be determined (82%) (Additional file 3). Other reasons for exclusions were 1) the studies did not fulfil the inclusion/exclusion criteria (14%) and 2) articles reporting results that were already published in other articles (i.e. duplicate publications) (4%). Thus, in all 85 articles were included in this review.

### Study characteristics

The included articles were published in 39 different journals of which 4 journals (Spine (26%), Rheumatology (18%), Annals of Rheumatic Diseases (18%), Arthritis & Rheumatism (15%)) accounted for approximately three quarters of all journals. There was an uneven distribution of publications between 2000 and 2011, but with no clear patterns across the decade. The majority of the studies were from Europe (58%) followed by Australasia (21%), North America (18%) and Middle East (4%).

### Risk of bias within each study and across studies

**Figure 2:**
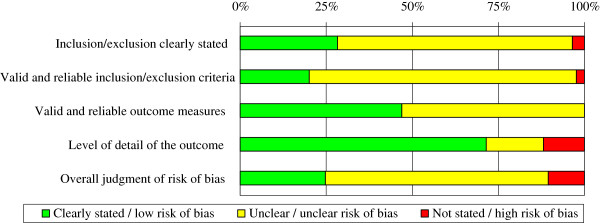
Risk of bias – Summary of all studies.

Overall, 25% of the studies were determined as having a low risk of bias and 11% were deemed as having a high bias risk (Figure 2 and Additional file 4). Thus, in approximately 65% of the studies it was unclear if risk of bias were either low or high, mainly because it was difficult to determine if the final study sample was truly representative of the target population. The risk of bias for each of the included studies is presented within each of the musculoskeletal conditions.

### Prevalence of musculoskeletal disorders in the elderly population

A total of 173 different prevalence estimates were extracted from the 85 included studies. The most commonly reported MSK condition (i.e. number of prevalence estimates) was BP (29%), OA and OP (17%), followed by RA (8%), ankle/foot pain (8%), knee pain (6%), hip pain (5%), shoulder pain (5%), hand/wrist pain (3%), and elbow pain (3%).

### Prevalence of RA

Rheumatoid arthritis was described in 12 studies with a total of 13 different point prevalence estimates [10-21] (Table 4). Seven (58%) were of low risk of bias [10,11,13,16,17,19,20] and only one study [18] was deemed as being of high risk of bias (Table 4 and Additional file 4).

**Table 4:** Description of studies on rheumatoid arthritis (RA)

**First author Publ. year Country**	**Study design / Population /Method of collection**	**Sample size**			**Crude response rate (%)**	**Outcome definition**	**Outcome assessment method**	**Prevalence period**	**Age**	Prevalence* (95% CI)			**Risk of bias**
		**Total**	**M**	**F**						**M**	**F**	**Total**	
Andrianakos [10] 2006 Greece	1966-99, (19+ yo), the total adult population in 7 mixed communities + random sample in another 2 mixed communities (the ESORDIG study). Home visit by a rheumatologist. Interview and examination	8740	4269	4471	82	ACR 1987 criteria	Self report + examination	Point	60-69			0.9	L
									70+			0.9	
Carmona [11] 2001 Spain	(20+ yo), a stratified multistage cluster sampling from the censuses of 20 municipalities. Postal questionnaire + interview by a rheumatologist	2192	1014	1178	73	ACR criteria, based on modified questionnaire	Self report	Point	60-69			1.0	L
									70-79			0.5	
									80+			2.7	
Collerton [12] 2009 UK	2006-7, (85+ yo), all people born in 1921, permanently registered with a participating GP in Newcastle upon Tyne or North Tyneside primary care trusts (the Newcastle 85+ Study). Medical records at the GP	853	323	530	59	Not stated	Medical record	Point	85	0.5	5.1	3.5	U
Englund [13] 2010 Sweden	2008, (20+ yo), all patients diagnosed with RA registered in the Skåne Health Care Register, southern Sweden. Data from a national registry	931316	27%	73%	N/A	Diagnosis of RA given by a specialist in rheumatology or internal medicine	National register	Point	65-74	1.0	1.9	1.5	L
									75-84	1.0	1.7	1.5	
									85+	1.2	1.2	1.1	
Hanova [14] 2006 Czech Republic	2002, (16+ yo), all patients diagnosed before 28th February reported by all rheumatologists, other specialists, and almost all GPs. Medical records from GPs.	?	?	?	N/A	ACR 1987 clinical criteria	Medical record	Point	60-69	0.6	2.3		U
									70-79	0.6	2.9		
									80+	0.5	0.8		
Laiho [15] 2001 Finland	1989, (75, 80 & 85 yo), a computer-generated random sample from the population register, Helsinki & Vantaa (the Helsinki Ageing Study & the Vantaa study). Interview and examination	1317	484	833	76-96	ACR 1987 clinical criteria	Self report + examination	Point	75	2.8	1.2	1.7	U
									80	0	1.4	1.0	
									85	0	1.3	1.0	
Neovius [16] 2010 Sweden	2008, (16+ yo), patients with a clinical visit listing an RA diagnosis were identified in inpatient and outpatient specialist care in the National Patient Register (1964–2007) together with patients listed in the Swedish Rheumatology Quality Register (SRQ; 1995–2007). National register	58102	?	?	?	Any visit listing an RA diagnosis was used to define RA.	National register	Point	60-69	0.9	2.1	1.5	L
									70-79	1.3	2.6	2.0	
									80+	1.5	2.7	2.2	
Ollivier [17] 2004 France	1996, (18+ yo), a random sample from the official list of phone numbers in Brittany. Telephone interviews by a rheumatologist	1672	0	1672	92	ACR 1987 clinical criteria	Self report + examination	Point	60-69		1.5		L
									70-79		1.1		
									80-89		1.4		
Picavet [18] 2003 The Netherlands	1998, (25+ yo), a 6 months follow-up on a baseline stratified random sample taken from the population register (the Dutch population-based Musculoskeletal Complaints and Consequences Cohort study, DMC3-study). Postal questionnaire	2338	?	?	85	"Please indicate whether a physician or medical specialist has ever told you that you have one or more of the following diseases [RA]”	Self repot	Point	65-74			6	H
									75+			10	
Rasch [19] 2003 USA	1988-94, (60+ yo), a multistage, stratified probability sample representative of the civilian non-institutionalized population residing in the 50 states of the USA. Home interviews and examination at mobile centers	5302	?	?	80	ACR 1987 clinical criteria: 3 out of 6 criteria met (“n of k”)	Self report + laboratory results	Point	60+	1.6 (0.8-2.4)	2.4 (1.4-3.4)		L
Rasch [19] 2003 USA	1988-94, (60+ yo), a multistage, stratified probability sample representative of the civilian non-institutionalized population residing in the 50 states of the USA. Home interviews and examination at mobile centers	5302	?	?	80	ACR 1987 clinical criteria: allowing surrogate classification variables when a primary classification variable is unavailable (“classification tree”)	Self report + laboratory results	Point	60+	1.6 (0.8-2.4)	2.6 (1.6-3.6)		L
Riise [20] 2000 Norway	1987 & 1996, (20+), all records of patients registered at the Department of Rheumatology at the University Hospital of Tromsø [only 1996 prevalence reported here]	2282	?	?	?	ACR 1987 clinical criteria (ICD-9 diagnoses 714.0 and 714.9) in medical records and subsequently critical reviews by a senior consultant	Medical record	Point	60-69	0.9	1.4	1.2	L
									70-79	0.9	1.9	1.5	
									80-89	1.3	1.3	1.5	
									90+	0.2	0.6	0.4	
Symmons [21] 2002 UK	(16+), a two-stage stratified random sample from 11 GPs in Norfolk (GPs allowed to exclude certain patients). Postal questionnaire and examination at the GP	5424	?	?	77	A modified version of the ACR 1987 criteria for symptomatic RA followed by a clinical assessment	Clinical assessment	Point	65-74	1.5 (0.8-3.0)	3.3 (1.9-5.9)		U
									75+	3.1 (1.7-5.5)	5.4 (3.1-9.3)		

*Prevalence estimates without decimals are obtained from figures/graphs in the article and should be interpreted with caution.

I: Interview, Q: Questionnaire; E: Examination, R: Register. L: Low, U: Unclear, H: High.

GP: General practitioner; ACR: The American College of Rheumatology (ACR clinical criteria for RA [22]).

The prevalence estimates that were based on clearly defined criteria (typically the 1987 American College of Rheumatology (ACR) criteria [22]) ranged between 0.4% and 2.2%. The prevalence of RA was higher among women. No clear age related differences could be determined, but generally the prevalences were minimal across ages.

### Prevalence of OA

Sixteen studies reported prevalence estimates on OA in four different anatomical sites (knee, hand, hip, and lumbar spine) either based on symptomatic findings only, radiographic findings only, or on a combination of both [11,18,23-36] (Table 5). Of these studies, five (31%) were judged as being of low risk [11,23-25,30] and only one study (6%) of high risk of bias [18] (Table 5 and Additional file 4).

**Table 5:** Description of studies on osteoarthritis (OA)

	**First author Publ. year Country**	**Study design / Population /Method of collection**	**Sample size**			**Crude response rate (%)**	**Outcome definition**	**Outcome assessment method**	**Prevalence period**	**Age**	Prevalence* (95% CI)			**Risk of bias**
			**Total**	**M**	**F**						**M**	**F**	**Total**	
Knee, symptomatic	Carmona [11] 2001 Spain	(20+ yo), a stratified multistage cluster sample from the censuses of 20 municipalities. Postal questionnaire + interview (rheumatologist)	2192	1014	1178	73	ACR clinical criteria	Self report	Point	60-69			28.1	L
										70-79			33.7	
										80+			21.3	
Knee, symptomatic	Fernandez-Lopez [25] 2008 Spain	2000, (20+ yo), stratified poly-stage cluster sampling from 20 city censuses, home visit questionnaire + interview (rheumatologist)	2192	1014	1178	73	ACR clinical criteria	Self report	Point	60-69	18.1	37.2	28.1	L
										70-79	16.7	44.1	33.7	
										80+	14.3	25.5	21.3	
Knee, symptomatic	Mannoni [30] 2003 Italy	1995, (65+ yo), the entire population of 65+ yo in Dicomano (The ICARe Dicomano study). Home interview and examination (geriatrician)	697	406	291	81	ACR clinical criteria	Clinical examination	Point	65+			29.8	L
Knee, symptomatic	Picavet [18] 2003 The Netherlands	1998, (25+ yo), a 6 months follow-up on a baseline stratified random sample taken from the population register (the DMC3-study). Postal questionnaire	2338	?	?	85	"Please indicate whether a physician or medical specialist has ever told you that you have one or more of the following diseases [OA]”	Self report	Point	65-74			27	H
										75+			28	
Knee, radiographic	Jordan [27] 2007 USA	1991-7, (45+ yo), stratified simple random sampling of streets as primary sampling units and stratified subsampling of Caucasian women age 65 years or older residents of one of 6 townships (the Johnston County Osteoarthritis Project). Home interview and clinical examination	3690	?	?	72	K-L ≥2	Radiograph	Point	65-74			36.1 (33.8-38.6)	U
										75+			49.9 (45.4-54.4)	
Knee, radiographic	Kim [28] 2010 South Korea	2007, (50+ yo), a follow-up study of a random proportional sample from the Korean National Census of elderly community residents in Chuncheon city. Home interview, Questionnaire and examination	504	230	274	55	K-L ≥2	Radiograph	Point	60-69	4	40	26	U
										70-79	18	65	42	
										80-89	34	98	65	
Knee, radiographic	Muraki [31] 2009 Japan	2002, (65+ yo), random samples of community-dwelling people from listings of resident registration in three communities (Itabashi-ku, Hidakagawa-cho, Taiji-cho). Interview, Questionnaire and examination	2282	817	1465	29-76	K-L ≥2	Radiograph	Point	65-69	42	61		U
										70-74	46	71		
										75-79	51	74		
										80+	53	81		
Knee, radiographic	Sudo [32] 2008 Japan	(65+ yo), all community inhabitants recruited in Miyagawa village, in central Mie Prefecture. Questionnaire and interview (hospital)	598	205	393	40	K-L ≥2	Radiograph	Point	65-74	14	33		U
										75-84	26	41		
										85+	23	47		
Knee, radiographic	Yoshida [34] 2002 Japan	2000, (40+ yo), all women identified by the municipal electroral list of Oshima town, Nagasaki (The Hizen-Oshima Study). examination	586		586	30	K-L ≥2	Radiograph	Point	63-69		35.8		U
										70-79		54.0		
										80-89		63.3		
Knee, radiographic	Yoshimura [33] 2009 Japan	2005-7, (40+ yo), recruited from the resident-registration lists of the Hidakagawa & Taiji regions or from a randomly selected cohort study from the Itabashi (Tokyo) Ward resident registration database (the ROAD study). Examination	3040	1061	1979	76	K-L ≥2	Radiograph	Point	60-69	35.2	57.1		U
										70-79	48.2	71.9		
										80+	51.6	80.7		
Knee, symptomatic + radiographic	Andrianakos [23] 2006 Greece	1966-99, (19+ yo), the total adult population in 7 mixed communities + random sample in another 2 mixed communities (the ESORDIG study). Interview, Questionnaire and examination (home visit, rheumatologist)	8740	4269	4471	82	ACR clinical criteria + radiograph (unknown definition)	Self report + radiograph	Point	60-64	5.4	21.4	13.3	L
										65-69	8.4	21.1	15.3	
										70-74	11.7	28.0	20.4	
										75-79	19.3	33.3	27.6	
										80+	27.2	27.2	22.5	
Knee, symptomatic + radiographic	Sudo [32] 2008 Japan	(65+ yo), all community inhabitants recruited in Miyagawa village, in central Mie Prefecture. Questionnaire and interview (hospital)	598	205	393	40	Questionnaire (no additional information) + K-L ≥2	Self report + radiograph	Point	65-74	8	26		U
										75-84	17	28		
										85+	16	31		
Knee, symptomatic + radiographic	Jordan [27] 2007 USA	1991-7, (45+ yo), stratified simple random sampling of streets as primary sampling units and stratified subsampling of Caucasian women age 65 years or older residents of one of 6 townships (the Johnston County Osteoarthritis Project). Home interview + clinical examination	3690	?	?	72	“On most days, do you have pain, aching, or stiffness in your (right, left) knee?” + K-L ≥2	Self report + radiograph	Point	65-74			20.8 28.8-23.0)	U
										75+			32.8 (29.5-36.3)	
Knee, symptomatic + radiographic	Kim [28] 2010 South Korea	2007, (50+ yo), a follow-up study of a random proportional sample from the Korean National Census of elderly community residents in Chuncheon city. Home interview, Questionnaire and examination	504	230	274	55	“Have you experienced pain, aching, or stiffness lasting at least a month in a knee?” + K-L grade ≥2	Self report + radiograph	Point	60-69	2	27	17	U
										70-79	9	48	28	
										80-89	12	63	38	
										76-94		18.7		
Knee, symptomatic + radiographic	Muraki [31] 2009 Japan	2002, (65+ yo), random samples of community-dwelling people from listings of resident registration in three communities (Itabashi-ku, Hidakagawa-cho, Taiji-cho). Interview, Questionnaire and examination	2282	817	1465	29-76	Knee pain lasting at least 1 month with pain having last occurred within the current or previous year + K-L ≥2	Self report + radiograph	Point	65-69	17	22		U
										70-74	15	36		
										75-79	16	34		
										80+	18	39		
Hand, symptomatic	Carmona [11] 2001 Spain	(20+ yo), a stratified multistage cluster sample from the censuses of 20 municipalities. Postal Questionnaire + Interview (rheumatologist)	2192	1014	1178	73	ACR clinical criteria	Self report	Point	60-69			15.3	L
										70-79			23.9	
										80+			17.3	
Hand, symptomatic	Mannoni [37] 2003 Italy	1995, (65+ yo), the entire population of 65+ yo in Dicomano (The ICARe Dicomano study). Home interview and examination (geriatician)	697	406	291	81	ACR clinical; criteria	Clinical examination	Point	65+			14.9	L
Hand, radiographic	Dillon [24] 2007 USA	1991-4, (60+ yo), a multistage, cluster and stratified representative sample of US civilians (NHANES III). Home Questionnaire and Interview, Examination in mobile examination centre	2498	?	?	62	NHANES III criteria, but with no history of persistent symptoms	Self report + clinical examination	Point	60-69			31.5	L
										70-79			43.9	
										80+			41.2	
Hand, radiographic	Haugen [26] 2011 Norway	1992-5 & 2002–5, (28–92 yo), baseline data from the 1992–5 Community cohort of the Framingham Heart Study selected through random-digit dialing and from the 2002–5 Offspring cohort, Massachusetts. Postal questionnaire + examination	2300	?	?	43	Modified K-L grade ≥2 (2 = mild HOA, i.e. small OP(s) and/or mild JSN, sclerosis may be present)	Radiograph	Point	60-64	56	63		U
										65-69	71	82		
										70-74	78	91		
										75-79	72	92		
										80+	96	100		
Hand, radiographic	Kwok [29] 2011 The Netherlands	1997-3, (55+ yo), responders from follow-up of 1990–3 random sample of inhabitants living in the Ommoord district, Rotterdam (the Rotterdam Study). Questionnaire and Interview (home), Examination	3430	1509	1921	43	‘Mild’ OA defined as KL grade ≥2 in at least one finger joint	Radiograph	Point	65-74	56.3	68.4	62.9	U
										75-84	63.3	78.9	72.8	
										85+	66.7	68.4	67.9	
Hand, symptomatic + radiographic	Andrianakos [23] 2006 Greece	1966-99, (19+ yo), the total adult population in 7 mixed communities + random sample in another 2 mixed communities (the ESORDIG study). Interview, Questionnaire and examination (home visit, rheumatologist).	8740	4269	4471	82	ACR clinical criteria + radiograph (unknown definition)	Self report + radiograph	Point	60-64	0.9	7.0	3.9	L
										65-69	2.1	8.8	5.7	
										70-74	3.3	7.8	5.8	
										75-79	4.0	8.1	6.5	
										80+	1.8	5.5	4.2	
Hand, symptomatic + radiographic	Dillon [24] 2007 USA	1991-4, (60+ yo), a multistage, cluster and stratified representative sample of US civilians (NHANES III). Home Questionnaire and Interview, Examination (mobile examination centre).	2498	?	?	62	NHANES III criteria	Self report + clinical examination	Point	60-69			6.1	L
										70-79			9.9	
										80+			9.7	
Hand, symptomatic + radiographic	Kwok [29] 2011 The Netherlands	1997-3, (55+ yo), responders from follow-up of 1990–3 random sample of inhabitants living in the Ommoord district, Rotterdam (the Rotterdam Study). Questionnaire and Interview (home), Examination	3430	1509	1921	43	‘Did you have any pain in the right or left hand during the last month?’ + ‘Mild’ OA defined as KL grade ≥2 in at least one finger joint	Self report + radiograph	Point	65-74	6.1	18.9	13.1	U
										75-84	5.3	14.2	10.7	
										85+	0.0	21.1	14.3	
Hand, symptomatic + radiographic	Zhang [36] 2002 USA	1992-3, (71+ yo), all participants from the original cohort in 1948 aged 26–62 (the Framingham Study). Questionnaire and Interview, Examination	1032	369	663	89	“On most days, do you have pain, aching, or stiffness in any of your joints?” + K-L ≥2	Self report + radiograph	Point	71-74	16.4	27.2		U
										75-79	11.9	26.1		
										80+	13.5	26.0		
Hip, symptomatic	Picavet [18] 2003 The Netherlands	1998, (25+ yo), a 6 months follow-up on a baseline stratified random sample taken from the population register (the DMC3-study). Postal questionnaire	2338	?	?	85	"Please indicate whether a physician or medical specialist has ever told you that you have one or more of the following diseases [OA]”	Self report	Life time	65-74			17	H
										75+			22	
Hip, symptomatic	Mannoni [37] 2003 Italy	1995, (65+ yo), the entire population of 65+ yo in Dicomano (The ICARe Dicomano study). Interview and examination (home visit, geriatrician)	697	406	291	81	ACR clinical criteria	Clinical examination	Point	65+			7.7	L
Hip, symptomatic + radiographic	Andrianakos [23] 2006 Greece	1966-99, (19+ yo), the total adult population in 7 mixed communities + random sample in another 2 mixed communities (the ESORDIG study). Interview, Questionnaire and examination (home visit, geriatrician)	8740	4269	4471	82	ACR clinical criteria + radiograph (unknown definition)	Self report + radiograph	Point	60-64	0.7	3.5	2.1	L
										65-69	0.5	4.1	2.4	
										70-74	1.2	3.9	2.6	
										75-79	0.6	4.3	3.0	
										80+	0.6	2.8	1.8	
Lumbar spine radiographic	Yoshimura [33] 2009 Japan	2005-7, (40+ yo), recruited from the resident-registration lists of the Hidakagawa & Taiji regions or from a randomly selected cohort study from the Itabashi (Tokyo) Ward resident registration database (the ROAD study). Examination	3040	1061	1979	76	K-L ≥3	Radiograph	Point	60-69	74.6	64.3		U
										70-79	85.3	76.1		
										80+	89.9	79.6		
Lumbar spine radiographic	Yoshimura [35] 2009 Japan	1990, (40-79yo), all inhabitants from the register of residents in Miyama village were invited (the Miyama Study). Examination	400	200	200	100	K-L ≥3	Radiograph	Point	60-69	39.6	38.0		U
										70-79	38.3	34.7		

*Prevalence estimates without decimals are obtained from figures/graphs in the article and should be interpreted with caution.

R: Register. L: Low, U: Unclear, H: High.

GP: General practitioner; ACR: The American College of Rheumatology (ACR clinical criteria for RA [22]).

#### Lumbar spine OA

Two Japanese studies on lumbar spine radiographic OA, using a higher Kellgren-Lawrence (K-L) grade (≥3), reported point prevalences of 40%-75% in the 60–69 year olds to 80%-90% in the 80+ age group [33,35].

#### Hip OA

Only three studies on hip OA were found in this review [18,23,37], two studies on symptomatic hip OA [18,37] and one on combined symptomatic/radiographic hip OA [23]. The self reported hip OA were about three times higher (17-22%) than found through clinical examination (approx. 8%) and more common in women than in men [23]. Combined symptomatic/radiographic hip OA increased from 2% in the 60–64 year olds to 3% in the 75–79 year olds, but then decreased slightly in the 80+ year olds.

#### Knee OA

Knee OA was reported in 11 studies [11,18,23,25,27,28,30-34] and presented 14 different prevalence estimates (Table 5). The ACR clinical criteria [38] for knee OA was used in two out of three studies on symptomatic knee pain and showed fairly similar prevalence estimates (28-33%).

All studies on radiographic knee OA only (i.e. without reported pain) either used the K-L grade 2 [39,40] or higher criteria for OA [27,28,31-34]. Nevertheless, great variations in point prevalence estimates were reported. For example, in women in their sixties, OA was present in 40% to 57%, and in the seventies it ranged between 54% and 74%. In men, larger differences were found (60s: 4%-35%) and (70s: 18%-51%). Overall, higher OA estimates were reported with increasing age.

For the combined knee OA and reported pain, generally larger gender differences were seen (Table 5) and more variation in age trends were also noted [23,27,28,31,32]. Painful knee OA increased with age until approximately at age 80+ where a slight decrease was reported in two out of the four studies [11,18,25,30].

#### Hand OA

Seven studies included data on hand OA [11,23,24,26,29,36,37] with a total of eight prevalence estimates on symptomatic [11,37], radiographic [26,29], and combined symptomatic/radiographic hand OA [23,24,29,36] (Table 5).

Regardless of hand OA definitions, women had more OA than men and overall, OA increased with age, although several studies also reported a slight decrease in the oldest age groups.

Five studies reported either symptomatic hand OA only [11,37] or radiographic hand OA only [24,26,29], all with different definitions and age ranges. Nevertheless, similar point prevalences were noted: Approximately 15% of the “younger” elderly population reported symptomatic hand OA. Radiographic hand OA ranged from approximately 56% in the “youngest” elderly men to 100% in the oldest women.

The point prevalence estimates of combined symptomatic/radiographic hand OA ranged from approximately 4% in the “youngest” elderly population to approximately 14% in the oldest people and were therefore less common than radiographic hand OA alone.

### Prevalence of OP

Twenty-one studies reported prevalence estimates on OP of which 14 studies measured the bone mineral density (BMD) in five well-defined anatomical areas (lumbar spine/hip, lumbar spine only, hip/femoral neck only, hand, and heel) [33,35,41-52]. Seven studies used other definitions and were mostly based on self reported data [12,18,53-57] (Table 6). Four studies (19%) were of high risk of bias [18,47,51,54], whereas only two studies (10%) were of low risk of bias [41,52] (Table 6 and Additional file 4).

**Table 6:** Description of studies on osteoporosis (OP)

	**First author Publ. year Country**	**Study design / Population /Method of collection**	**Sample size**			**Crude response rate (%)**	**Outcome definition**	**Outcome assessment method**	**Prevalence period**	**Age**	Prevalence* (95% CI)			**Risk of bias**
			**Total**	**M**	**F**						**M**	**F**	**Total**	
Lumbar spine or hip	Andrianakos [41] 2006 Greece	1966-99, (19+ yo), the total population in 7 mixed communities + random sample in another 2 mixed communities. Examination (rheumatological centers)	8740	4269	4471	82	WHO BMD T-score −2.5 SD or less	DXA	Point	59-64			7	L
										69+			10	
Lumbar spine or hip	Bleicher [43] 2010 Australia	2005-07, (70+ yo), community-dwelling in three local government areas around Sydney (CHAMP). Questionnaire + Examination	1626	1626	0	45	Pharmaceutical Benefits Scheme criteria for OP: BMD T-score −3 SD or less	Hologic DXA	Point	70-74			5.0	U
										75-79			4.0	
										80-84			5.0	
										85-89			5.0	
										90+			14.0	
Lumbar spine or hip	Naves [48] 2005 Spain	(50+ yo), randomly selected from the Oviedo municipal register. Postal questionnaire + examination	229	229	0	74	The Int. Society of Clinical Densitometry: BMD with a T-score −2.5 SD or less	Hologic DXA, QDR 1000 densitometer	Point	80+	12.5			U
Lumbar spine or hip	Sanfélix-Genovés [49] 2010 Spain	2006-7, (50+ yo), stratified random sample of women included in the Population Information System of the Valencia Healthcare Agency, Valencia (the FRAVO Study). Interview, questionnaire + examination	824	0	824	47	WHO BMD T-score −2.5 SD or less	Norland & Hologic densitometer	Point	60-64		22.5 (16.3-28.8)		U
										65-69		32.4 (25.2-39.4)		
										70-74		39.9 (31.8-47.9)		
										75+		49.3 (37.4-61.2)		
Lumbar spine or hip	Vestergaard [52] 2005 Denmark	1995-9, all in- and outpatients recorded in The National Hospital Discharge Register (=100%) based on all ICD-10 codes on OP. National register	11359	1426	9933	N/A	WHO BMD T-score −2.5 SD or less	The National Hospital Discharge Register	Point	60-64	14.7		29.6	L
										65-69	19.9		44.0	
										70-74	26.1		59.1	
										75-79	33.1		72.2	
										80-84	40.4		81.3	
										85-89	47.8		85.8	
										90-94	55.3		88.6	
										95+	64.3		92.3	
Lumbar spine	Cui [44] 2008 South Korea	2004-5, (20–79 yo), from the Namwon study and the Thyroid Disease Prevalence study and from two provinces. Interview + questionnaire + clinical examination	4148	1810	2338	39	WHO BMD T-score −2.5 SD or less	Lunar DXA	Point	60-69	8.7	51.3		U
										70-79	12.8	60.2		
Lumbar spine	Henry [45] 2000 Australia	1997, (20–94 yo), age-stratified, random, population-based sample of women registered (compulsory) in the Commonwealth of Australia Electoral Rolls, Geelong. Questionnaire + examination.	1494	0	1494	63	WHO BMD T-score −2.5 SD or less	Lunar DXA, DPX-L densitometer	Point	60-64		10.5		U
										65-69		15.2		
										70-79		28.8		
										80+				
Lumbar spine	Sanfélix-Genovés [49] 2010 Spain	2006-7, (50+ yo), stratified random sample of women included in the Population Information System of the Valencia Healthcare Agency, Valencia (the FRAVO Study). Interview, questionnaire + examination	824	0	824	47	WHO BMD T-score −2.5 SD or less	Norland & Hologic densitometer	Point	60-64		18.5 (12.7-24.3)		U
										65-69		28.2 (21.4-35.0)		
										70-74		37.8 (28.8-45.7)		
										75+		39.1 (27.5-50.7)		
Lumbar spine	Shin [50] 2010 South Korea	2006-7, (40+ yo), selected group from the 2001 cohort of residents in the farming community of Ansung through mailing, door-to-door and telehpone solicitations (the Korean Health and Genome Study, KHGS). Examination	3538	1547	1991	71	WHO BMD T-score −2.5 SD or less	Lunar Prodigy DXA	Point	60-69	13.7	28.5		U
										70-79	22.4	47.5		
Lumbar spine	Vestergaard [52] 2005 Denmark	1995-9, all in- and outpatients recorded in The National Hospital Discharge Register (=100%) based on all ICD-10 codes on OP. Register (National)	11359	1426	9933	N/A	WHO BMD T-score −2.5 SD or less	The National Hospital Discharge Register	Point	60-64	3.4		17.3	L
										65-69	4.6		27.7	
										70-74	6.0		39.6	
										75-79	7.9		51.1	
										80-84	10.1		60.2	
										85-89	12.7		66.0	
										90-94	15.8		68.2	
										95+	19.8		65.6	
Lumbar spine	Yang [51] 2004 Taiwan	1994-8, female patients entering a hospital for a DXA scan. Examination (hospital)	4689	0	4689	?	Threshold level, lumbar spine < 0.827 g/cm2	Lunar DXA	Point	60-69		14.1		H
										70-79		14.3		
										80+		16.1		
Lumbar spine	Yoshimura [35] 2009 Japan	1990, (40–79 yo), all inhabitants from the register of residents in Miyama village (the Miyama Study). Examination	400	200	200	100	WHO BMD T-score −2.5 SD or less	Lunar DXA	Point	60-69	12.0	38.0		U
										70-79	14.0	60.0		
Lumbar spine	Yoshimura [33] 2009 Japan	2005-7, (40+ yo), recruited from the resident-registration lists of the Hidakagawa & Taiji regions or from a randomly selected cohort study from the Itabashi (Tokyo) Ward resident registration database (the ROAD study). Examination	3040	1061	1979	76	Criteria of the Japanese Society of Bone and Mineral Research (BMD <70% of PBM: lumbar spine BMD < 0.708 g/cm2)	Hologic DXA	Point	60-69	2.6	13.5		U
										70-79	3.6	29.8		
										80+	7.4	43.8		
Hip	Vestergaard [52] 2005 Denmark	1995-9, all in- and outpatients recorded in The National Hospital Discharge Register (=100%) based on all ICD-10 codes on OP. National register	11359	1426	9933	N/A	WHO BMD T-score −2.5 SD or less	The National Hospital Discharge Register	Point	60-64	12.7	20.0		L
										65-69	17.7	30.4		
										70-74	23.7	42.5		
										75-79	30.8	54.6		
										80-84	38.6	65.4		
										85-89	46.9	73.9		
										90-94	55.3	79.9		
										95+	64.3	83.9		
Femoral neck	Cui [44] 2008 South Korea	2004-5, (20–79 yo), from the Namwon study and the Thyroid Disease Prevalence study invited to clinical examination and interview, from two provinces. Interview, questionnaire + examination	4148	1810	2338	39	WHO BMD T-score −2.5 SD or less	Lunar DXA	Point	60-69	7.3	11.4		U
										70-79	15.2	36.7		
Femoral neck	Henry [45] 2000 Australia	1997, (20–94 yo), age-stratified, random, population-based sample of women registered (compulsory) in the Commonwealth of Australia Electoral Rolls, Geelong. Questionnaire + examination	1494	0	1494	63	WHO BMD T-score −2.5 SD (NB. Hip: femoral neck used in this review)	Lunar DXA, DPX-L densitometer	Point	60-64		15.2		U
										65-69		20.8		
										70-79		31.6		
										80+		36.5		
Femoral neck	Holt [46] 2002 UK	(50+ yo), random sample from seven health centres (Aberdeen, Bath, rural Cambridgeshire, Harrow, Truro, Norfolk, and Cambridge City). Questionnaire + examination	7426	2253	5173	48	WHO BMD T-score −2.5 SD (NB. Hip: femoral neck used in this review)	Hologic DXA, QDR 1000 densitometer	Point	65+	2.7	8.1		U
Femoral neck	Sanfélix-Genovés [49] 2010 Spain	2006-7, (50+ yo), stratified random sample of women included in the Population Information System of the Valencia Healthcare Agency, Valencia (the FRAVO Study). Interview, questionnaire + examination	824	0	824	47	WHO BMD T-score −2.5 SD or less	Norland & Hologic Densitometer.	Point	60-64	6.9 (3.1-10.7)			U
										65-69	10.1 (9.4-21.3)			
										70-74	15.4 (9.4-21.3)			
										75+	34.8 (23.4-46.1)			
Femoral neck	Yang [51] 2004 Taiwan	1994-8, female patients entering a hospital for a DXA scan. Examination (hospital)	4689	0	4689	?	Threshold level, femoral neck < 0.605 g/cm2.	Lunar DXA	Point	60-69		11.2		H
										70-79		17.3		
										80+		24.0		
Femoral neck	Yoshimura [33] 2009 Japan	2005-7, (40+ yo), recruited from the resident-registration lists of the Hidakagawa & Taiji regions or from a randomly selected cohort study from the Itabashi (Tokyo) Ward resident registration database (the ROAD study). Examination	3040	1061	1979	76	Criteria of the Japanese Society of Bone and Mineral Research (BMD <70% of PBM): femoral neck < 0604 g/cm2 (men) & < 0.55 g/cm2 (women)	Hologic DXA	Point	60-69	7.0	22.2		U
										70-79	22.3	42.9		
										80+	13.0	65.1		
Phalanges	Biino [42] 2011 Italy	2003-2008, (30–103 yo), all residents from 10 villages of the Ogliastra region, Sardinia. Interview + examination	6326	2024	4302	51	AD-SoS T-score −3.2 SD or less	Quantitative II-V phalanges ultrasound	Point	60-69	9.6	24.2		U
										70-79	13.6	42.7		
										80+	25.8	62.1		
Heel	Kenny [47] 2009 USA	Community-dwelling and assisted living adults recruited through community talks. Questionnaire + examination	114	81	33	?	BMD T-score level not stated	Lunar QUS, Quantitative heel Ultrasound	Point	82.4 ± 4.6			31.6	H
Other	Cheng [53] 2009 USA	1999-2005, (65+ yo), a 5% national sample from beneficiaries fee-for service Medicare parts A and B coverage, not enrolled in a health maintenance organisation. Register (Medicare)	911327	359733	551594	N/A	Beneficiaries with at least one claim for certain OP related services and with ICD code for OP or fractures associated with OP	ICD-9 code for OP	Point	65-69			2.0	U
										70-74			17.2	
										75-79			25.5	
										80+			55.4	
Other	Collerton [12] 2009 UK	2006-7, (85+ yo), all people born in 1921, permanently registered with a participating GP in Newcastle upon Tyne or North Tyneside primary care trusts (the Newcastle 85+ Study). Register (GP)	853	323	530	59	Not stated	Medical record	Point	85	3.8	20.0	14.2	U
Other	Kotz [54] 2004 USA	1994 + 1995, (16–94 yo at baseline in 1965), responders who have survived until at least 1994, from the random representative sample of women from the Alameda County Study, California. Postal questionnaire	1171	0	1171	97	Ever had osteoporosis?	Self report	Life time	66-75		11.5		H
										76-94		18.7		
Other	Lespessailles [55] 2009 France	2006, (45+ yo), stratified random sample of women from the national population data (INSEE) (the INSTANT study). Interview + questionnaire (door to door)	2613	0	2613	N/A	Whether they had osteoporosis + whether this had been diagnosed by bone densitometry	Self report	Life time	60-64		10		U
										65-69		14		
										70-74		17		
										75-79		16		
										80-84		15		
										85+		10		
Other.	Picavet [18] 2003 The Netherlands	1998, (25+ yo), a 6 months follow-up on a baseline stratified random sample taken from the population register (the DMC3-study). Postal questionnaire	2338	?	?	85	"Please indicate whether a physician or medical specialist has ever told you that you have one or more of the following diseases [OP]”	Self report	Life time	65-74			13	H
										75+			15	
Other.	Saks [56] 2001 Estonia	2000, (65+ yo), a stratified random sample of patients from 200 random GPs in 16 Estonian regions. Register (GP).	811	391	420	81	GP diagnosis without any further description or validation	Medical record	Point	65-84			15.2	U
										85+			19.5	
Other	Werner [57] 2003 Israel	1997-8, (60+ yo), a stratified random sample of Jewish and Arab community-dwelling persons from the Central Bureau of Statistics (the Israeli Survey of Elderly Persons Aged 60 and Over Study). Interview + questionnaire (home visit)	3022	1688	1334	60	Whether a physician had ever diagnosed them as having osteoporosis	Self report	Life time	60-69	5.0	20.5		U
										70-79	8.0	26.3		
										80+	7.9	28.3		

*Prevalence estimates without decimals are obtained from figures/graphs in the article and should be interpreted with caution.

R: Register. L: Low, U: Unclear, H: High.

BMD: Bone mineral density; WHO: World Health Organization, GP: general practitioner, DXA: Dual X-ray absorptometry.

Regardless of the anatomical site, a steady increase in OP with increasing age for all types of OP definitions was seen. Generally, OP was two-three times more common in women than in men.

#### Lumbar spine OP

Eight studies included data on lumbar OP [33,35,44,45,49-52], all using the WHO BMD T-score of −2.5 SD or less [58], except for two studies [33,51] (Table 6). While the Spanish and Danish OP age related prevalences in women were similar (ranging 17%-66%), greater age related variations were noted in women in the Asian countries. For example, in South Korean women, markedly higher estimates across ages (51%-61%) were reported by Cui et al. [44] compared to Shin et al. (29%-48%) [50].

#### Hip or femoral neck OP

Seven studies reported either hip or femoral neck OP [33,44-46,49,51]. Fairly similar results were noted in South Korea and Australia (range: 11%-37% for 60–79 year olds) [44,45], but the UK and Spanish estimates were slightly lower (range: 7%-15% for 60–74 year olds) [46,49].

#### Combined lumbar spine and/or hip OP

Lumbar spine and/or hip OP was reported in five studies [41,43,48,49,52] which all, except for one study [43], used the WHO bone mineral density (BMD) threshold (T-score) of −2.5 SD or less (Table 6). The prevalence of OP was slightly higher in Danish women [52] (range: 30%-92%) than in Spanish women [49] (range: 23%-49%).

### Prevalence of BP

In all, BP 31 studies were included [11,41,59-87] of which seven (23%) studies were of low risk of bias [11,41,73-75,78,80] and three (10%) of high risk of bias [59,81,83] (Table 7 and Additional file 4).

**Table 7:** Description of back pain (BP) and neck pain (NP)

	**First author Publ. year Country**	**Study design / Population / Method of collection**	**Sample size**			**Crude response rate (%)**	**Outcome definition**	**Outcome assessment method**	**Prevalence period**	**Age**	Prevalence* (95% CI)			**Risk of bias**
			**Total**	**M**	**F**						**M**	**F**	**Total**	
LBP	Andrianakos [41] 2006 Greece	1966-99, (19+ yo), the total population in 7 mixed communities + random sample in another 2 mixed communities. Interview, questionnaire + examination (home visit, rheumatologist)	8740	4269	4471	82	LBP localized in the back area between the lower limits of the chest and the gluteal folds, either radiating or not along a lower extremity. Past LBP included if recurrent and chronic causes	Self report	Life time	59-64			18	L
										69+			19	
LBP	Salaffi [79] 2005 Italy	2004, (18+ yo), stratified randomised sample selected from the practice lists of 16 general practitioner-GPs representative of the practices in the Marches, central Italy. Postal questionnaire	2155	?	?	54	LBP defined as pain localized in the back area between the lower limits of the chest and the gluteal folds, either radiating or not along a lower extremity. Three satisfactory screening criteria: I) Report of ever having had LBP, II) A health care provider visit for LBP in the previous six months, and III) LBP that began more than 3 months previously	Self report	Life time	65-74			29	U
										75+			26	
LBP	Cecchi [60] 2006 Italy	1998-2000, (65+), a representative cohort was selected from the registries of Greve in Chianti (rural area) and Bagno a Ripoli (urban area near Florence). Interview, questionnaire + examination (home visit, rheumatologist).	1008	443	565	80	Any frequent BP episodes (defined as quite often-almost every day) over the past 12 months	Self report	One year	65-74	20.7	38.1		U
										75-84	26.3	44.4		
										85+	25.0	25.0		
LBP	Hartvigsen [69] 2006 Denmark	2003, (70–102 yo), twins from the populations-based twin study (LSADT). Interview + questionnaire (home)	1844	?	?	84	Modified version of the standardised Nordic Questionnaire (SNQ) on Musculoskeletal Pain	Self report	One year	72-102	21 (19–23)	32 (29–35)		U
LBP	Hicks [71] 2008 USA	(62+ yo), community-dwellers from 4 retirement communities (The Retirement Community Back Pain Study). Postal questionnaire	522	170	352	52	“In the past year, have you had any low back pain? If yes, please rate your usual back pain over the past year on a scale from 0 to 10"	Self report	One year	60-69			26.7	U
										70-79			30.5	
										80+			24.8	
LBP	Picavet [78] 2003 The Netherlands	1998, (25+ yo), stratified random sample taken from the population register (the DMC3-study). Postal questionnaire	3664	45%	55%	46	"Did you have pain [in the lower part of the back] during the past 12 months?"	Self report	One year	65-74			48	U
										75+			32	
LBP	Santos-Eggimann [80] 2000 Switzerland	1992-3, (25-74 yo), two-stage probabilistic stratified random sample of inhabitants from the population files of the Vaud-Fribourg & Ticino communes (the WHO MONICA study). Questionnaire (postal) + examination.	3227	?	?	61	The Standardized Nordic Questionnaire: any ache, pain, or discomfort located in the lower back (indicated by the shaded area on a diagram), with or without radiation to one or both legs (sciatica) the preceding 12 months	Self report	One year (>7 days)	65-74	28.5	38.5		L
LBP	Goubert [66] 2004 Belgium	2001, (17+ yo), a representative access panel of individuals who regularly participate in postal surveys. Postal questionnaire	1624	?	?	65	Participants indicated whether they had experienced LBP pain in the past six months (The Graded Chronic Pain Scale)	Self report	Six months	65+			36.7	U
LBP	Miro [75] 2007 Spain	(65+ yo), stratified random sample taken from the population census obtained from the Catalan Statistics Institute, Catalonia. Interview + questionnaire (local primary care centre).	592	274	318	99	The Chronic Pain Grade: “In the past 3 months have you had pain that has lasted for one day or longer in any part of your body?”	Self report	Three months	65-74			61.0	L
										75-84			62.6	
										85+			44.2	
LBP	Parsons [77] 2007 UK	2001-3, (18+ yo), random samples from 16 Medical Research Council General Practice Research Framework practices, South East quadrant of the UK. Postal questionnaire	2501	1347	1154	47	The Chronic Pain Grade: Any ‘pain which has lasted for 3 months or longer and currently troubles respondents either all of the time or on and off'	Self report	Three months	65-74			7	U
										75-101			6	
LBP	Strine [82] 2007 USA	2002, (18+ yo), Multistage cluster sample of random households from all 50 states and DC (the NHIS). Interview + questionnaire (home).	29828	?	?	96	“During the past 3 months did you have LBP [lasting a whole day or more and not fleeting or minor]?” [NB. LBP only, NP not included]	Self report	Three months	65+			19.7	U
													(18.4-20.9)	
LBP	Hartvigsen [69] 2005 Denmark	2003, (70–102 yo), twins from the populations-based twin study (LSADT). Interview + questionnaire (home).	1844	?	?	84	Modified version of the standardised Nordic Questionnaire (SNQ) on Musculoskeletal Pain	Self report	One month	72-102	20 (17–23)	30 (27–33)		U
LBP	Meyer [74] 2007 USA	1998-2000, (65+ yo), follow-up of a random sample of members from a random sample of 269 Medicare + Choice plans (the HOS) (NB. Only 2000 data reported here). Questionnaire (home) + interview (phone).	55690	?	?	61	“In the past 4 wk, how often has low back pain interfered with your usual daily activities? (work, school or housework)”	Self report	One month	65+			49.4	L
LBP	Stranjalis [81] 2004 Greece	2000, (15+ yo), a 2000 person sample, selected via a multi-stage sampling of rural, semi-urban and urban residents through "random numbers" of starting points followed by "statistical step of five" in 47 cities, towns or villages (until reaching a total of 2000 persons). Interview + questionnaire (home).	1846	?	?	92	"Did you have low back pain during the last month?"	Self report	One month	65+			46.9	H
LBP	Suka [83] 2009 Japan	2005, About 1000 persons from five different healthcare facilities were asked to participate. Questionnaire (Health care facility).	5652	?	?	?	Musculoskeletal pain for more than 1 week during the last month (marked on a drawing with predefined body regions)	Self report	One month	60-69	23.8	23.2		H
LBP	Thomas [87] 2004 UK	(50+ yo), all patients from three GPs from the North Staffordshire Primary Care Research Consortium (the NorStOP). Postal questionnaire	7878	?	?	70	“In the past 4 weeks have you had pain that has lasted for one day or longer in any part of your body?” [supplemented by a full body manikin]	Self report	One month	60-69			35.1	U
										70-79			29.9	
										80+			27.3	
LBP	Webb [85] 2003 UK	(16+ yo), stratified sample of patients from three GP in West Pennine, East of Manchester. Questionnaire.	4515	?	?	78	Pain lasting for more than 1 week, over the last month, in any of seven areas (back, neck, shoulder, elbow, hand, hip, knee) or in multiple joints	Self report	One month	65-74	20.6	32.1		U
										75+	17.4	30.9		
LBP	Yaron [86] 2011 Israel	2002, 2006, 2008, (20+ yo), stratified sample drawn from a telephone database on different population sectors. Telephone interview + questionnaire	2520	47%	53%	59-66	The Community Oriented Program for the Control of Rheumatic Diseases core questionnaire (CCQ): “In the past 7 days have you experienced pain in any of the following sites: [ankles]?”	Self report	One week	61+			67.2	U
LBP	Baek [59] 2010 South Korea	2005-06, (65+ yo), residents of Seongnam City. Questionnaire + examination (hospital).	714	299	415	64	The Oswestry Disability Index on LBP: "pain at the moment"	Self report	Point	65-69			70.1	H
										70-74			70.3	
										75-79			81.3	
										80+			70.5	
LBP	Carmona [11] 2001 Spain	(20+ yo), a stratified multistage cluster sample from the censuses of 20 municipalities. Questionnaire (home) + interview (rheumatologist).	2192	1014	1178	73	LBP defined by self-report. The interviewers were instructed to indicate what was understood by low back and then to ask about pain in that area	Self report	Point	60-69			21.2	L
										70-79			12.3	
										80+			4.0	
LBP	Freburger [64] 2009 USA	1992 + 2006, (21+ yo), two-staged stratified probability sample of North Carolina households with telephone numbers (NB only data from 2006 survey is included). Interview + questionnaire (phone).	2723	?	?	83	LBP defined as pain at the level of the waist or below, with or without buttock and/or leg pain. Chronic LBP: 1) pain and activity limitations nearly every day for the past 3 months or 2) more than 24 episodes of pain that limited activity for 1 day or more in the past year	Self report	Point	65+			12.3	U
LBP	Picavet [78] 2003 The Netherlands	1998, (25+ yo), stratified random sample taken from the population register (the DMC3-study). Postal questionnaire	3664	45%	55%	46	[Lower part of the back] pain during the survey	Self report	Point	65+	23.3 (19.8-26.8)	29.5 (25.8-33.2)		L
														
BP	Denard [62] 2010 UK	2000-2, (65+ yo), a random sample of 300 community dwelling men recruited at 6 US academic medical centers (The MrOS cohort). Questionnaire (postal) + examination.	300	295	0	98	Any BP in the past 12 months	Self report	One year	65+	65			U
BP	Keenan [73] 2006 UK	1993, (55+ yo), a two-stage random sample from the North Yorkshire Family Health Services Authority. Postal questionnaire	16222	?	?	86	Any swelling, pain, or stiffness in any of their joints that lasted >6 weeks in the previous 3 months (identified on a manikin)	Self report	Three months	65-74	13.5 (12.2-14.8)	18.2 (16.8-19.7)	16.1 (14.7-17.5)	L
										75+	11.4 (10.2-12.6)	19.0 (17.6-20.5)	16.4 (15.1-17.8)	
BP	Hartvigsen [68] 2004 Denmark	1995,1997,1999, 2001, (70–102 yo), twins from the populations-based twin study (LSADT). Interview + questionnaire (home).	4484	?	?	100	“Have you during the past month suffered from pain or stiffness in the neck or shoulders?” + diagnosis had been made by a physician	Self report	One month	70-74	14	18		U
										75-79	12	17		
										80-84	10	15		
										85+	11	16		
BP	Hartvigsen [70] 2008 Denmark	2005, (100 yo), all Danes born in 1905 were located through the Danish Civil Registration System. Interview + questionnaire (home).	256	?	?	56	“During the past month, have you been suffering from back pain, acute back pain, or lumbago?”	Self report	One month	100	16.7	29.4	27.3	U
BP	Docking [63] 2011 UK	1988-90, (75+ yo), original cohort from the 1985 Cambridge City over 75 s Cohort Study randomly chosen from a selection of geographically and socially representative general practices in Cambridge. Interview + questionnaire (home).	1174	35%	65%	45%	Have you recently had an illness or condition which prevented you carrying out normal day to day routine? [accompanied by a list of conditions including back pain]. (=Any back pain)	Self report	Point	77-79			27.0	U
										80-84			31.1	
										85-89			27.0	
										90-100			29.1	
BP	Jacobs [72] 2006 Jerusalem	1990 & 1998–9, (70 & 77 yo), recruited from the electoral register of the Israeli Ministry of Interior by their serial number’s last digit, West Jerusalem. Questionnaire (home) + examination (hospital).	277	?	?	60	Subjects were asked if they have back pain. Further questions on the duration, frequency, site, and severity of their pain. Chronic BP was defined as reporting pain on a frequent basis	Self report	Point	70			44	U
										77			58	
NP	Andrianakos [41] 2006 Greece	1966-99, (19+ yo), the total population in 7 mixed communities + random sample in another 2 mixed communities. Interview, questionnaire + examination (home visit, rheumatologist)	8740	4269	4471	82	NP localized in the neck either radiating or not along an upper extremity	Self report	Life time	59-64			9	L
										69+			8	
NP	Chiu [61] 2006 HongKong	2001, (15+ yo), residents selected through a two-stage randomization process. Interview + questionnaire (phone).	664	295	364	66	“Up to the present time, have you ever had neck pain?” + "at least once in the past 12 months" + "within the past 7 days"	Self report	One year	65+			9.3	U
NP	Hartvigsen [69] 2006 Denmark	2003, (70–102 yo), twins from the populations-based twin study (LSADT). Interview + questionnaire (home).	1844	?	?	84	Modified version of the standardised Nordic Questionnaire (SNQ) on Musculoskeletal Pain	Self report	One year	72-102	16 (13–19)	20 (18–22)		U
NP	Vogt [84] 2003 USA	1997-8, (70-79yo), a random sample of age-eligible white Medicare beneficiaries from lists provided by the Health Care Financing Administra-tion and all age-eligible black community residents in designated zip code areas close to the Pittsburgh, PA, and Memphis, TN, field centers (the Health ABC study). Interview + examination												
(home).	3075	1491	1584	?	Neck or shoulder pain lasting at least 1 month during the previous year	Self report	One year	70-79			11.9 (10.8-13.0)	U		
NP	Keenan [73] 2006 UK	1993, (55+ yo), a two-stage random sample from the North Yorkshire Family Health Services Authority. Postal questionnaire	16222	?	?	86	Any swelling, pain, or stiffness in any of their joints that lasted >6 weeks in the previous 3 months. (identified on a manikin)	Self report	Three months	65-74	13.1 (11.8-14.4)	17.3 (16.0-18.7)	15.4 (14.1-16.8)	L
										75+	10.6 (9.4-11.8)	16.7 (15.3-18.1)	14.6 (13.3-15.9)	
NP	Miro [75] 2007 Spain	(65+ yo), stratified random sample taken from the population census obtained from the Catalan Statistics Institute, Catalonia. Interview + questionnaire (local primary care centre)	592	274	318	99	The Chronic Pain Grade: “In the past 3 months have you had pain that has lasted for one day or longer in any part of your body?”	Self report	Three months	65-74			52.6	L
										75-84			56.4	
										85+			53.5	
NP	Parsons [77] 2007 UK	2001-3, (18+ yo), random samples from 16 Medical Research Council General Practice Research Framework practices, South East quadrant of the UK. Postal questionnaire	2501	1347	1154	47	The Chronic Pain Grade: Any ‘pain which has lasted for 3 months or longer and currently troubles respondents either all of the time or on and off'	Self report	Three months	65-74			5	L
										75-101			3	
NP	Strine [82] 2007 USA	2002, (18+ yo), Multistage cluster sample of random households from all 50 states and DC (the NHIS). Interview + questionnaire (home).	29828	?	?	96	“During the past 3 months did you have neck pain [lasting a whole day or more and not fleeting or minor]?” [NB. NP only, LBP not included]	Self report	Three months	65+			4.8 (4.4-5.2)	U
NP	Hartvigsen [68] 2004 Denmark	1995,1997,1999, 2001, (70–102 yo), twins from the populations-based twin study (LSADT). Interview + questionnaire (home).	4484	?	?	100	“Have you during the past month suffered from pain or stiffness in the neck or shoulders?” + diagnosis had been made by a physician	Self report	One month	70-74	11	9		U
										75-79	12	11		
										80-84	11	14		
										85+	10	11		
NP	Hartvigsen [69] 2006 Denmark	2003, (70–102 yo), twins from the populations-based twin study (LSADT). Interview + questionnaire (home).	1844	?	?	84	Modified version of the standardised Nordic Questionnaire (SNQ) on Musculoskeletal Pain	Self report	One month	72-102	19 (16–22)	24 (22–27)		U
NP	Hartvigsen [70] 2008 Denmark	2005, (100 yo), all Danes born in 1905 were located through the Danish Civil Registration System. Interview + questionnaire (home).	256	?	?	56	“During the past month, have you been suffering from stiffness or pain in the neck or shoulders?”	Self report	One month	100	19.1	22.6	22.1	U
NP	Thomas [87] 2004 UK	(50+ yo), all patients from three GPs from the North Staffordshire Primary Care Research Consortium (the NorStOP). Postal questionnaire	7878	?	?	70	“In the past 4 weeks have you had pain that has lasted for one day or longer in any part of your body?” [supplemented by a full body manikin]	Self report	One month	60-69			22.9	U
										70-79			17.7	
										80+			14.9	
NP	Webb [85] 2003 UK	(16+ yo), stratified sample of patients from three GP in West Pennine, East of Manchester. Questionnaire.	4515	?	?	78	Pain lasting for more than 1 week, over the last month, in any of seven areas (back, neck, shoulder, elbow, hand, hip, knee) or in multiple joints	Self report	One month	65-74	16.7	23.9		U
										75+	17.8	21.3		
NP	Natvig [76] 2004 Norway	1994, (24–76 yo), all inhabitants in six birth cohorts in Ullensaker municipality, northeast of Oslo. Postal questionnaire	3325	1501	1824	54	Standardised Nordic Questionnaire: Any pain or discomfort from the neck during the previous week (illustrated on a body mannequin)	Self report	One week	64-66			32.3	U
										74-76/ 84-86			24.1	
NP	Yaron [86] 2011 Israel	2002, 2006, 2008, (20+ yo), stratified sample drawn from a telephone database on different population sectors. Telephone interview + questionnaire	2520	47%	53%	59-66	The Community Oriented Program for the Control of Rheumatic Diseases core questionnaire (CCQ): “In the past 7 days have you experienced pain in any of the following sites: [ankles]?”	Self report	One week	61+			53.3	U
NP	Goode [65] 2010 USA	2006, (21+ yo), stratified random probability sample of North Carolina telephone numbers, USA. Interview + questionnaire (phone).	2809	?	?	86	“Neck discomfort or pain. Neck pain starts in the neck area; it may spread to the shoulder or arm.” Chronic, impairing NP 1) pain and activity limitations nearly every day for the past 3 months or 2) greater than 24 episodes of pain in the previous year, with each episode limiting activity for 1 day or more	Self report	Point	65+			1.2	U
NP	Guez [67] 2002 Sweden	1999, (25–74 yo), stratified randomised sample of inhabitants, mainly along the coastal area, northern Sweden (WHO MONICA Study). Questionnaire + examination (medical center)	6000	?	?	72	”Have you visited a doctor because of a neck or head injury?”, chronic NP defined as continuous neck complaints for more than 6 months	Self report	Point	65-74	18	20		U
NP	Picavet [78] 2003 The Netherlands	1998, (25+ yo), stratified random sample taken from the population register (the DMC3-study). Postal questionnaire	3664	45%	55%	46	[Neck] pain during the survey	Self report	Point	65+	17.3 (14.2-20.4)	25.0 (21.5-28.5)		L
Thoracic pain	Miro [75] 2007 Spain	(65+ yo), stratified random sample taken from the population census obtained from the Catalan Statistics Institute, Catalonia. Interview + questionnaire (local primary care centre)	592	274	318	99	The Chronic Pain Grade: “In the past 3 months have you had pain that has lasted for one day or longer in any part of your body?”	Self report	Three months	65-74			15.0	L
										75-84			12.9	
										85+			11.6	
Thoracic pain	Parsons [77] 2007 UK	2001-3, (18+ yo), random samples from 16 Medical Research Council General Practice Research Framework practices, South East quadrant of the UK. Postal questionnaire	2501	1347	1154	47	The Chronic Pain Grade: Any ‘pain which has lasted for 3 months or longer and currently troubles respondents either all of the time or on and off'	Self report	Three months	65-74			2	U
										75-101			2	
Higher back	Picavet [78] 2003 The Netherlands	1998, (25+ yo), stratified random sample taken from the population register (the DMC3-study). Postal questionnaire	3664	45%	55%	46	[Higher part of the back] pain during the survey	Self report	Point	65+	2.8 (1.4-4.2)	11.9 (9.2-14.6)		L

*Prevalence estimates without decimals are obtained from figures/graphs in the article and should be interpreted with caution.

R: Register. L: Low, U: Unclear, H: High.

#### Low back pain

Low back pain was reported in 20 studies all with different LBP definitions and with eight different prevalence periods (Table 7) [11,41,59,60,64,66,69,71,74,75,77-83,85-87].

The one-month prevalence was the most common prevalence period reported and ranged between 27% and 49%. The lowest estimates were based on more restricted definitions, whereas the larger estimates (47-49%) had less restricted LBP definitions.

Overall, the prevalence estimates increased up to 80 years of age and then dropped slightly after that. With one exception [83], women reported LBP more often than men.

#### Back pain

Back pain was used in six studies [62,63,68,70,72,73] on five different prevalence estimates, all with different BP definitions and with a wide range in prevalence estimates. Thus, one-month BP prevalence ranged between 18% and 29%, and the point prevalence ranged from 27% to 58%. Interestingly, in two studies where 100 year olds were included, the point and one-month BP was roughly the same (27%-29%) [63,70]. Prevalence estimates were all higher among women, but age-related changes are inconclusive as most studies did not demonstrate any major changes across ages.

#### Neck pain

Sixteen studies on NP reported six different prevalence periods [41,61,67-70,73,75-78,82,84-87] of which the one-month prevalence was the most commonly used period. No identical NP definitions were used and/or different age intervals were reported, although some definitions and intervals were fairly similar.

Overall, the one year prevalence ranged between 9% and 12% [41,61,71,84]. Greater variations were noted for the three-month prevalence, ranging between 5% [77] and 56% [75] in 65–74 year olds. Of the four one-month prevalence estimates using fairly similar NP definitions, about 23% reported NP [70,76,85,87]. Men reported NP less often than women and in all studies there was a decrease in NP with increasing age, albeit small in some studies.

#### Mid back pain

Finally, MBP (i.e. thoracic or higher back pain) was reported in three studies [75,77,78]. The three-month prevalence was used in two studies, but with different MBP definitions and thus, the prevalence ranged between 2% [77] and 15% [75]. One study showed that pain in the “higher back” was four times more prevalent among women [78].

### Prevalence of shoulder pain

Six studies reported five different prevalence periods on shoulder pain [73,77,78,84,86,88] and two studies also included upper arm pain using two different prevalence periods [87,89] (Table 8). Two studies (25%) were rated as having low risk of bias [73,77,78] and the rest as having an “unclear” risk of bias (Table 8 and Additional file 4).

**Table 8:** Description of studies on shoulder pain

	**First author Publ. year Country**	**Study design / Population /Method of collection**	**Sample size**			**Crude response rate (%)**	**Outcome definition**	**Outcome assessment method**	**Prevalence period**	**Age**	Prevalence* (95% CI)			**Risk of bias**
			**Total**	**M**	**F**						**M**	**F**	**Total**	
Shoulder pain	Hill [88] 2010 Australia	2004-6, (18+ yo), recruited randomly from the electronic White Pages telephone listings (the NWAH Study). Phone interview + questionnaire	3488	1712	1776	81	Ever had pain or aching in their shoulder at rest or when moving, on most days for at least a month	Self report	Life time	65-74			23.7	U
										75+			26.5	
Shoulder pain	Vogt [84] 2003 USA	1997-8, (70-79yo), a random sample of age-eligible white Medicare beneficiaries from lists provided by the Health Care Financing Administration and all age-eligible black community residents in designated zip code areas close to the Pittsburgh, PA, and Memphis, TN, field centers (the Health ABC study). Interview + examination (home)	3075	1491	1584	?	neck or shoulder pain lasting at least 1 month during the previous year	Self report	One year	70-79			18.9 (17.5-20.3)	U
Shoulder pain	Keenan [73] 2006 UK	1993, (55+ yo), a two-stage random sample from the North Yorkshire Family Health Services Authority. Postal questionnaire	16222	?	?	86	Any swelling, pain, or stiffness in any of their joints that lasted >6 weeks in the previous 3 months. (identified on a manikin)	Self report	Three months	65-74	12.6 (11.3-13.8)	17.9 (16.5-19.4)	15.5 (14.2-16.8)	L
										75+	13.1 (11.2-14.3)	21.0 (19.5-22.4)	18.3 (16.8-19.7)	
Shoulder pain	Parsons [77] 2007 UK	2001-3, (18+ yo), random samples from 16 Medical Research Council General Practice Research Framework practices, South East quadrant of the UK. Postal questionnaire	2501	1347	1154	47	The Chronic Pain Grade: Any ‘pain which has lasted for 3 months or longer and currently troubles respondents either all of the time or on and off'	Self report	Three months	65-74			4	U
										75-101			3	
Shoulder pain	Yaron [86] 2011 Israel	2002, 2006, 2008, (20+ yo), stratified sample drawn from a telephone database on different population sectors. Telephone Interview + questionnaire	2520	47%	53%	59-66	The Community Oriented Program for the Control of Rheumatic Diseases core questionnaire (CCQ): “In the past 7 days have you experienced pain in any of the following sites: [shoulders]?”	Self report	One week	61+			50.9	U
Shoulder pain	Picavet [78] 2003 The Netherlands	1998, (25+ yo), stratified random sample taken from the population register (the DMC3-study). Postal questionnaire	3664	45%	55%	46	[Shoulder] pain during the survey	Self report	Point	65+	13.2 (10.4-16.0)	23.1 (19.6-26.6)		L
Shoulder /upper arm pain	Gummesson [89] 2003 Sweden	1997, (25–74 yo), stratified randomised sample from the Swedish population register in southern Sweden. Postal questionnaire	2466	?	?	82	Chronic pain: ‘Where is the pain, numbness, or tingling located and since when have you had the symptoms?’ [shoulder/upper arm, since 3 months]	Self report	Point	65-74	10.3	19.9		U
Shoulder /upper arm pain	Thomas [87] 2004 UK	(50+ yo), all patients from three GPs from the North Staffordshire Primary Care Research Consortium (the NorStOP). Postal questionnaire	7878	?	?	70	“In the past 4 weeks have you had pain that has lasted for one day or longer in any part of your body?” [supplemented by a full body manikin]	Self report	One month	60-69			33.0	U
										70-79			28.0	
										80+			24.9	

*Prevalence estimates without decimals are obtained from figures/graphs in the article and should be interpreted with caution.

R: Register. L: Low, U: Unclear, H: High.

All studies used different shoulder pain definition and/or different prevalence periods. Nevertheless, in some of the studies with different prevalence periods, the estimates varied only slightly (3-5%) (65–74 year olds, men: 10%-13%; women: 18%-23%) [73,78,89]. In three studies where gender estimates were provided, women reported more pain than men [73,78,89]. Only one study provided different age intervals, which showed that shoulder pain increased slightly with age.

### Prevalence of elbow pain

Elbow pain was reported in four studies [73,77,78,86] and elbow/forearm pain in one study [89], of which three different prevalence periods were used (Table 9). Two studies (40%) were of low risk of bias [73,77,78], and the rest being unclear (Table 9 and Additional file  4).

**Table 9:** Description of studies on elbow pain

	**First author Publ. year Country**	**Study design / Population /Method of collection**	**Sample size**			**Crude response rate (%)**	**Outcome definition**	**Outcome assessment method**	**Prevalence period**	**Age**	Prevalence* (95% CI)			**Risk of bias**
			**Total**	**M**	**F**						**M**	**F**	**Total**	
Elbow pain	Keenan [73] 2006 UK	1993, (55+ yo), a two-stage random sample from the North Yorkshire Family Health Services Authority. Postal questionnaire	16222	?	?	86	any swelling, pain, or stiffness in any of their joints, that lasted >6 weeks in the previous 3 months (identified on a manikin)	Self report	Three months	65-74	4.6 (4.0-5.7)	6.4 (5.4-7.4)	5.7 (4.8-6.6)	L
										75+	4.4 (3.5-5.2)	8.3 (7.3-9.4)	7.0 (6.0-8.0)	
Elbow pain	Parsons [77] 2007 UK	2001-3, (18+ yo), random samples from 16 Medical Research Council General Practice Research Framework practices, South East quadrant of the UK. Postal questionnaire	2501	1347	1154	47	The Chronic Pain Grade: Any ‘pain which has lasted for 3 months or longer and currently troubles respondents either all of the time or on and off'	Self report	Three months	65-74		1	U	U
										75-101		2		
Elbow pain	Yaron [86] 2011 Israel	2002, 2006, 2008, (20+ yo), stratified sample drawn from a telephone database on different population sectors. Telephone interview + questionnaire	2520	47%	53%	59-66	The Community Oriented Program for the Control of Rheumatic Diseases core questionnaire (CCQ): “In the past 7 days have you experienced pain in any of the following sites: [elbow]?”	Self report	One week	61+			33.0	U
Elbow pain	Picavet [78] 2003 The Netherlands	1998, (25+ yo), stratified random sample taken from the population register (the DMC3-study). Postal questionnaire	3664	45%	55%	46	[Elbow] pain during the survey	Self report	Point	65+	4.9 (3.1-6.7)	8.0 (5.8-10.2)		L
Elbow/ forearm pain	Gummesson [89] 2003 Sweden	1997, (25–74 yo), stratified randomised sample from the Swedish population register in southern Sweden. Postal questionnaire	2466	?	?	82	Chronic pain: ‘Where is the pain, numbness, or tingling located and since when have you had the symptoms?’ [elbow/forearm, since 3 months]	Self report	Point	65-74	1.7	8.3		U

*Prevalence estimates without decimals are obtained from figures/graphs in the article and should be interpreted with caution.

I: Interview, Q: Questionnaire; E: Examination, R: Register. L: Low, U: Unclear, H: High.

GP: General practitioner; ACR: The American College of Rheumatology (ACR clinical criteria for RA [22]).

Different elbow pain definitions were used in each study. Nevertheless, similar estimates were reported for both point and three-month prevalences [73,78]. Thus, approximately 5% of men and 6%-8% of women reported elbow pain. Elbow pain increased with age [73,77]. Fewer men reported elbow pain compared to women [73,78].

### Prevalence of hand/wrist pain

Two studies reported hand pain only [73,87], one study wrist pain only [77], and three studies on combined wrist/hand pain [78,86,89] (Table 10). Two studies (33%) were of low risk of bias [73,77,78], and the rest were unclear (Table 10 and Additional file 4).

**Table 10:** Description of studies on wrist and hand pain

	**First author Publ. year Country**	**Study design / Population / Method of collection**	**Sample size**			**Crude response rate (%)**	**Outcome definition**	**Outcome assessment method**	**Prevalence period**	**Age**	Prevalence* (95% CI)			**Risk of bias**
			**Total**	**M**	**F**						**M**	**F**	**Total**	
Hand pain	Keenan [73] 2006 UK	1993, (55+ yo), a two-stage random sample from the North Yorkshire Family Health Services Authority. Postal questionnaire	16222	?	?	86	Any swelling, pain, or stiffness in any of their joints that lasted >6 weeks in the previous 3 months. (identified on a manikin)	Self report	3 months	65-74	14.2 (13.0-15.6)	23.3 (21.8-24.9)	19.2 (17.8-20.6)	L
										75+	11.6 (10.4-12.8)	25.3 (23.7-26.8)	20.6 (19.1-22.1)	
Hand pain	Thomas [87] 2004 UK	(50+ yo), all patients from three GPs from the North Staffordshire Primary Care Research Consortium (the NorStOP). Postal questionnaire	7878	?	?	70	“In the past 4 weeks have you had pain that has lasted for one day or longer in any part of your body?” [supplemented by a full body manikin]	Self report	One month	60-69			25.6	U
										70-79			20.2	
										80+			16.9	
Wrist pain	Parsons [77] 2007 UK	2001-3, (18+ yo), random samples from 16 Medical Research Council General Practice Research Framework practices, South East quadrant of the UK. Postal questionnaire	2501	1347	1154	47	The Chronic Pain Grade: Any ‘pain which has lasted for 3 months or longer and currently troubles respondents either all of the time or on and off'	Self report	3 months	65-74			4	U
										75-101			3	
Wrist/hand pain	Yaron [86] 2011 Israel	2002, 2006, 2008, (20+ yo), stratified sample drawn from a telephone database on different population sectors. Telephone interview + questionnaire	2520	47%	53%	59-66	The Community Oriented Program for the Control of Rheumatic Diseases core questionnaire (CCQ): “In the past 7 days have you experienced pain in any of the following sites: [hands/wrists]?”	Self report	One week	61+			33.0	U
Wrist/ hand pain	Gummesson [89] 2003 Sweden	1997, (25–74 yo), stratified randomised sample from the Swedish population register in southern Sweden. Postal questionnaire	2466	?	?	82	Chronic pain: ‘Where is the pain, numbness, or tingling located and since when have you had the symptoms?’ [wrist/hand, since 3 months]	Self report	Point	65-74	2.1	14.9		U
Wrist/ hand pain	Picavet [78] 2003 The Netherlands	1998, (25+ yo), stratified random sample taken from the population register (the DMC3-study). Postal questionnaire	3664	45%	55%	46	[Wrist/hand] pain during the survey	Self report	Point	65+	9.7 (7.3-12.1)	22.5 (19.1-25.9)		L

*Prevalence estimates without decimals are obtained from figures/graphs in the article and should be interpreted with caution.

L: Low, U: Unclear, H: High.

GP: General practitioner; ACR: The American College of Rheumatology (ACR clinical criteria for RA [22]).

Wrist and/or hand pain prevalence estimates varied greatly among the different studies. For example, as few as 14% of men aged 75+ [73] and as many as 26% of women aged 60–69 [87] reported hand pain. Also, 2% of men between 65–74 [89] and 22.5% of women (65+) [78] reported wrist/hand pain. Women reported more often wrist and/or hand pain than men [73,78,89]. Hand pain increased slightly with age in one study [73], but decreased in the other study [87].

### Prevalence of hip pain

Five different prevalence periods on hip pain were reported in nine studies [73,75,77,78,83,87,90-92] (Table 11). Three studies (33%) were considered to be of low risk of bias [73,75,78] and only one study (11%) of high risk of bias [83] (Table 11 and Additional file 4).

**Table 11:** Description of studies on hip pain

**First author Publ. year Country**	**Study design / Population /Method of collection**	**Sample size**			**Crude response rate (%)**	**Outcome definition**	**Outcome assessment method**	**Prevalence period**	**Age**	Prevalence* (95% CI)			**Risk of bias**
		**Total**	**M**	**F**						**M**	**F**	**Total**	
Peat [92] 2006 UK	2002, (50+ yo), all community-dwelling adults registered with 3 general practices in North Staffordshire (The NorStOP). Postal questionnaire	2429	1005	1424	22	The Regional Pains Survey, containing the Western Ontario & McMaster Universities Osteoarthritis Index on hip pain (the WOMAC-HIP)	Self report	One year	65-74	47	50		U
									75+	44	48		
Dawson [91] 2004 UK	2002, (65+), a random sample from the Oxfordshire Health Authority register. Postal questionnaire	3341	1557	1784	61	"During the past 12 months, have you had pain in or around either of your hips on most days for one month or longer?"	Self report	One year	65-74	14.7	23.1		U
									75-84	18.0	20.7		
									85+	18.8	21.0		
Keenan [73] 2006 UK	1993, (55+ yo), a two-stage random sample from the North Yorkshire Family Health Services Authority. Postal questionnaire	16222	?	?	86	Any swelling, pain, or stiffness in any of their joints that lasted >6 weeks in the previous 3 months (identified on a manikin)	Self report	3 months	65-74	10.2 (9.1-11.4)	14.4 (13.1-15.8)	12.5 (12.3-13.8)	L
									75+	7.3 (6.3-8.4)	17.2 (15.8-18.6)	13.8 (12.6-15.1)	
Miro [75] 2007 Spain	(65+ yo), stratified random sample taken from the population census obtained from the Catalan Statistics Institute, Catalonia. Interview + questionnaire (local primary care centre)	592	274	318	99	The Chronic Pain Grade: “In the past 3 months have you had pain that has lasted for one day or longer in any part of your body?”	Self report	3 months	65-74			30.3	L
									75-84			31.5	
									85+			30.2	
Parsons [77] 2007 UK	2001-3, (18+ yo), random samples from 16 Medical Research Council General Practice Research Framework practices, South East quadrant of the UK. Postal questionnaire	2501	1347	1154	47	The Chronic Pain Grade: Any ‘pain which has lasted for 3 months or longer and currently troubles respondents either all of the time or on and off'	Self report	3 months	65-74			5	U
									75-101			4	
Christmas [90] 2002 USA	1988-92 & 1991–4, (60+ yo), a multistage, cluster and stratified representative sample of US civilians (NHANES III). Home Questionnaire and Interview, Examination in mobile examination centre	6596	?	?	?	Significant hip pain on most days over the preceding 6 weeks	Self report & clinical examination	6 week	60-69	11	14		U
									70-79	12	17		
									80+	11	16		
Suka [83] 2009 Japan	2005, about 1000 persons from five different healthcare facilities were asked to participate. Questionnaire (Health care facility)	5652	?	?	?	Musculoskeletal pain (marked on a drawing with predefined body regions) for more than 1 week during the last month	Self report	One month	60-69	2.4	5.6		H
Thomas [87] 2004 UK	(50+ yo), all patients from three GPs from the North Staffordshire Primary Care Research Consortium (the NorStOP). Postal questionnaire	7878	?	?	70	“In the past 4 weeks have you had pain that has lasted for one day or longer in any part of your body?” [supplemented by a full body manikin]	Self report	One month	60-69			28.3	U
									70-79			27.0	
									80+			25.6	
Picavet [78] 2003 The Netherlands	1998, (25+ yo), stratified random sample taken from the population register (the DMC3-study). Postal questionnaire	3664	44.8%	55.2%	46	[Hip] pain during the survey	Self report	Point	65+	11.1 (8.5-13.7)	21.2 (17.8-24.5)		L

*Prevalence estimates without decimals are obtained from figures/graphs in the article and should be interpreted with caution.

I: Interview, Q: Questionnaire; E: Examination, R: Register. L: Low, U: Unclear, H: High.

All nine studies used different hip pain definitions, resulting in a wide prevalence range. For example, the three-month prevalence ranged between 5% and 30% in the elderly aged 65–74 [73,75,77]. Six studies reported gender specific prevalence estimates, all of which reported a higher prevalence in women [73,78,83,90-92]. Age related changes were somewhat unclear and only showed small (2-4%) differences across age groups.

### Prevalence of knee pain

Eleven studies reported five different prevalence periods on knee pain [27,73,77,78,83,86,87,91-94] (Table 12). Three studies (27%) were of low risk of bias [73,78,94] and one study being of high risk of bias [83] (Table 12 and Additional file 4).

**Table 12:** Description of studies on knee pain

**First author Publ. year Country**	**Study design / Population / Method of collection**	**Sample size**			**Crude response rate (%)**	**Outcome definition**	**Outcome assessment method**	**Prevalence period**	**Age**	Prevalence* (95% CI)			**Risk of bias**
		**Total**	**M**	**F**						**M**	**F**	**Total**	
Dawson [91] 2004 UK	2002, (65+), a random sample from the Oxfordshire Health Authority register. Postal questionnaire	3341	1557	1784	61	"During the past 12 months, have you had pain in or around either of your hips on most days for one month or longer?"	Self report	One year	65-74	26.1	36.2		U
									75-84	31.0	37.4		
									85+	32.3	35.5		
Jinks [94] 2008 UK	(50+), all pxatients registered at three general practices in North Staffordshire. Postal questionnaire	2059	?	?	56	Have had pain in or around either knee in the last 12 months (NB. Only ‘severe’ pain can be extracted from “new onset” of knee pain)	Self report	One year	65-74			8	L
									75+			12	
Peat [92] 2006 UK	2002, (50+ yo), all community-dwelling adults registered with 3 general practices in North Staffordshire (The NorStOP). Postal questionnaire	2429	1005	1424	22	The Regional Pains Survey, containing the Western Ontario & McMaster Universities Osteoarthritis Index on hip pain (the WOMAC-KNEE)	Self report	One year	65-74	70	71		U
									75+	62	74		
Keenan [73] 2006 UK	1993, (55+ yo), a two-stage random sample from the North Yorkshire Family Health Services Authority. Postal questionnaire	16222	?	?	q	Any swelling, pain, or stiffness in any of their joints that lasted >6 weeks in the previous 3 months. (identified on a manikin)	Self report	Three months	65-74	18.7 (17.3-?)	24.2 (22.6-25.7)	21.7 (20.2-23.2)	L
									75+	17.4 (16.0-18.8)	31.2 (29.5-32.8)	26.4 (24.9-28.0)	
Parsons [77] 2007 UK	2001-3, (18+ yo), random samples from 16 Medical Research Council General Practice Research Framework practices, South East quadrant of the UK. Postal questionnaire	2501	1347	1154	47	The Chronic Pain Grade: Any ‘pain which has lasted for 3 months or longer and currently troubles respondents either all of the time or on and off'	Self report	Three months	65-74			6	U
									75-101			6	
Croft [93] 2005 UK	(50+), all patients registered at three general practices in North Staffordshire	5346	45%	55%	59	‘Draw on a blank body manikin any pain or ache that had lasted for ≥1 day in the last month’	Self report	One month	65-74			63.4	U
									75+			60.4	
Suka [83] 2009 Japan	2005, about 1000 persons from five different healthcare facilities were asked to participate. Interview + questionnaire (Health care facility)	5652	?	?	?	Musculoskeletal pain (marked on a drawing with predefined body regions) for more than 1 week during the last month	Self report	One month	60-69	8.8	15.7		H
Thomas [87] 2004 UK	(50+ yo), all patients from three GPs from the North Staffordshire Primary Care Research Consortium (the NorStOP). Postal questionnaire	7878	?	?	70	“In the past 4 weeks have you had pain that has lasted for one day or longer in any part of your body?” [supplemented by a full body manikin]	Self report	One month	60-69			37.7	U
									70-79			35.4	
									80+			37.6	
Yaron [86] 2011 Israel	2002, 2006, 2008, (20+ yo), stratified sample drawn from a telephone database on different population sectors. Telephone interview + questionnaire	2520	47.2%	52.8%	59-66	The Community Oriented Program for the Control of Rheumatic Diseases core questionnaire (CCQ): “In the past 7 days have you experienced pain in any of the following sites: [knees]?”	Self report	One week	61+			63.9	U
Jordan [27] 2007 USA	1991-7, (45+ yo), stratified simple random sampling of streets as primary sampling units and stratified subsampling of Caucasian women age 65 years or older residents of one of 6 townships (the Johnston County Osteoarthritis Project). Home interview + clinical examination (local clinic)	3690	?	?	72	“On most days, do you have pain, aching, or stiffness in your knee?”	Self report	Point	65-74			49 (46.1-51.9)	U
									75+			56.6 (52.7-60.4)	
Picavet [78] 2003 The Netherlands	1998, (25+ yo), stratified random sample taken from the population register (the DMC3-study). Postal questionnaire	3664	44.8%	55.2%	46	[Knee] pain during the survey	Self report	Point	65+	16.2 (13.2-19.2)	27.6 (23.9-31.3)		L

*Prevalence estimates without decimals are obtained from figures/graphs in the article and should be interpreted with caution.

I: Interview, Q: Questionnaire; E: Examination, R: Register. L: Low, U: Unclear, H: High.

All 11 studies used different pain definitions which resulted in great variations in prevalence estimates. For example, in the 65–74 year olds, the one-year prevalence varied between 26% and 70% in men and between 36% and 71% [91,92]. Generally, there was an increase in knee pain with increasing age, ranging between 3% and 8% [27,73,92,94]. Some studies reported a slight decrease [91,93] whereas others found no change with increasing age [77,87]. Five studies included gender specific prevalences and all showed that more women than men reported knee pain [73,78,83,91,92].

### Prevalence of ankle/foot pain

Nine studies included information on foot pain [73,75,78,87,92,95-98], three studies on ankle pain [78,86,99], and one study on both ankle/foot pain [77] (Table 13). Of these 12 studies in total, five (42%) were of low risk of bias [73,75,78,96,98] and only one study was considered being of high risk of bias [97] (Table 13 and Additional file 4).

**Table 13:** Description of studies on ankle and foot pain

	**First author Publ. year Country**	**Study design / Population / Method of collection**	**Sample size**			**Crude response rate (%)**	**Outcome definition**	**Outcome assessment method**	**Prevalence period**	**Age**	Prevalence* (95% CI)			**Risk of bias**
			**Total**	**M**	**F**						**M**	**F**	**Total**	
Ankle pain	Dunn [99] 2004 UK	2001-2, (65+ yo), individuals born on or before July 31, 1935 and residing in Springfield, identified by Medicare beneficiary files and the Springfield town census. Interview + examination (home)	784	339	445	10	pain or discomfort in any of their joints on most days during the past 4 weeks	Self report	One month	75+	14.1	15.3	14.9	U
Ankle pain	Yaron [86] 2011 Israel	2002, 2006, 2008, (20+ yo), stratified sample drawn from a telephone database on different population sectors. Telephone interview + questionnaire	2520	47.2%	52.8%	59-66	The Community Oriented Program for the Control of Rheumatic Diseases core questionnaire (CCQ): “In the past 7 days have you experienced pain in any of the following sites: [ankles]?”	Self report	One week	61+			35.9	U
Ankle pain	Picavet [78] 2003 The Netherlands	1998, (25+ yo), stratified random sample taken from the population register (the DMC3-study). Postal questionnaire	3664	44.8%	55.2%	46	[Ankle] pain during the survey	Self report	Point	65+	4.6 (2.9-6.3)	9.8 (7.4-12.2)		L
Ankle/foot pain	Parsons [77] 2007 UK	2001-3, (18+ yo), random samples from 16 Medical Research Council General Practice Research Framework practices, South East quadrant of the UK. Postal questionnaire	2501	1347	1154	47	The Chronic Pain Grade: Any ‘pain which has lasted for 3 months or longer and currently troubles respondents either all of the time or on and off'	Self report	Three months	65-74			4	U
										75-101			5	
Foot pain	Peat [92] 2006 UK	2002, (50+ yo), all community-dwelling adults registered with 3 general practices in North Staffordshire (The NorStOP). Postal questionnaire	2429	1005	1424	22	The Regional Pains Survey, containing the Foot Disability Index (the FDI-FOOT)	Self report	One year	65-74	45	58		U
										75+	51	55		
Foot pain	Keenan [73] 2006 UK	1993, (55+ yo), a two-stage random sample from the North Yorkshire Family Health Services Authority. Postal questionnaire	16222	?	?	86	Any swelling, pain, or stiffness in any of their joints that lasted >6 weeks in the previous 3 months (identified on a manikin)	Self report	Three months	65-74	14.1 (12.8-15.4)	20.7 (19.2-22.2)	17.7 (16.3-19.1)	L
										75+	14.0 (12.7-15.3)	26.9 (25.3-28.3)	22.5 (21.0-24.0)	
Foot pain	Miro [75] 2007 Spain	(65+ yo), stratified random sample taken from the population census obtained from the Catalan Statistics Institute, Catalonia. Interview + questionnaire (local primary care centre)	592	274	318	99	The Chronic Pain Grade: “In the past 3 months have you had pain that has lasted for one day or longer in any part of your body?”	Self report	Three months	65-74			37.4	L
										75-84			44.1	
										85+			55.8	
Foot pain	Mickle [97] 2010 Australia	(60+ yo), from 16 randomly selected federal electorates in Sydney and Illawarra statistical regions, New South Wales. Questionnaire	312	158	154	16	The Manchester Foot Pain and Disability Index (MFPDI) ≥ 1	Self report	One month	60+			50	H
Foot pain	Mølgaard [98] 2010 Denmark	2005, (18–80 yo), random sample from the Danish Civil Registration System of the Aalborg municipality. Postal questionnaire	1671	807	864	80	"Have you within the last month had pain in your feet which lasted more than one day?"	Self report	One month	60-80			28.6	L
Foot pain	Thomas [87] 2004 UK	(50+ yo), all patients from three GPs from the North Staffordshire Primary Care Research Consortium (the NorStOP). Postal questionnaire	7878	?	?	70	“In the past 4 weeks have you had pain that has lasted for one day or longer in any part of your body?” [supplemented by a full body manikin]	Self report	One month	60-69			23.5	U
										70-79			22.5	
										80+			19.5	
Foot pain	Badlissi [95] 2005 USA	2001-2, (65+ yo), individuals born on or before July 31, 1935 and residing in Springfield, identified by Medicare beneficiary files and the Springfield town census. Interview + questionnaire (telephone) + examination (home visit)	784	339	445	10	Aches or pains in your feet past week or any foot pain or discomfort on most days during the past four weeks	Self report	One month	65+			41.6	H
Foot pain	Menz [96] 2005 Australia	(62–92 yo), combined independent units and serviced apartments in retirement village. Questionnaire + examination (home)	176	56	120	?	Subjects were asked whether they suffered from painful feet	Self report	Point	62-92	14	28	24	L
Foot pain	Picavet [78] 2003 The Netherlands	1998, (25+ yo), stratified random sample taken from the population register (the DMC3-study). Postal questionnaire	3664	44.8%	55.2%	46	[Foot] pain during the survey	Self report	Point	65+	8.9 (6.3-11.2)	11.8 (9.2-14.4)		L

*Prevalence estimates without decimals are obtained from figures/graphs in the article and should be interpreted with caution.

I: Interview, Q: Questionnaire; E: Examination, R: Register. L: Low, U: Unclear, H: High.

Two studies with similar designs and definitions reported that 23%-29% of 60–80 year olds had pain in their feet during the past month [87,98]. In contrast, two other similar studies on point prevalence showed greater variations (65+ men: 9%-14%; women: 12%-28%) [78,96]. Otherwise, great variations in prevalence were found, for the same reasons as described under the wrist/hand pain section. In all the studies reporting gender prevalences, women suffered more from ankle and/or foot pain than men [73,78,92,96,99]. In two studies, foot pain increased with age [73,75], but dropped in another study [87].

### Musculoskeletal co-morbidity

Information on multiple/widespread MSK conditions in the elderly population was extracted from 15 studies [30,59,68,72,75,78,82,84,86,87,89,91-93,100].

In a Danish elderly population (70–120 year olds), concurrent neck and BP was found in 13% of women and 8% of men [68]. The same findings were reported in the USA, where 9% of 65+ year olds had both NP and LBP [82]. Jacobs et al. reported an almost two-fold increase in concurrent joint pain among older people (70 and 77 year olds) with chronic BP (59% and 74% respectively) compared to those without chronic BP [72].

Widespread pain was reported in the study by Natvig et al., where 14-15% of Norwegian people aged 64–86 years had additional MSK pain (from either shoulders, elbows, hands/wrists, upper back, lower back, hips, knees, or ankles/feet) [100]. In Sweden, between 4% and 6% of men aged 65–74 with upper extremity pain also reported either NP, LBP, or lower extremity pain, whereas in women the reported prevalence was about three times higher (15%-17%) [89]. According to Vogt et al., 14% of 70 to 79 year old Americans reported concurrent MSK pain in at least four sites [84]. In the UK, three studies on multiple pain sites showed varying results among 65+ year olds, which may be due to different definitions [91-93]. According to Dawson et al., 11% of the older adults had both hip and knee pain [91]. Croft et al. reported slightly higher estimates (26%-33%) but included the whole body [93]. In the study by Peat et al., 40% had more than one painful joint in the lower extremity [92]. More widespread pain (up to 44 pain sites) was reported by 12%-16% of women and by 7%-13% of men aged 60 and over [87]. In Italy, “polyarticular peripheral joint pain” was reported in 28% in the same age group (65+) [30]. In a Dutch study, multiple MSK pain sites were present in roughly 28% of men and in 46% of women aged 65 and over [78].

Other studies report several MSK pain sites in more than half of the elderly people, which indicates overlapping MSK symptoms [59,75,86]. In a South Korean elderly population (65+), more than half reported both upper extremity pain as well as LBP and/or lower extremity pain [59]. Similarly, in an Israeli population of elderly people aged 61 and over, more than half reported LBP, NP, knee and shoulder pain [86]. Furthermore, at least a third of these people also reported other peripheral joint pain sites. Finally, in a Spanish study, people aged 65 and over had on average four MSK pain sites [75]. Unfortunately, it is not possible to determine how many of these suffered from multiple pain sites. Thus, based on these three studies, a high degree of overlapping/concurrent MSK pain sites must be present [59,75,86].

### In summary

The prevalence of MSK conditions remains high even in old age regardless of the type of complaint.

Women typically report problems more often than men, regardless of the MSK condition.

The prevalence of MSK complaints typically drops slightly in the oldest age group (i.e. 80+ year olds), except for OP where all studies report an age related increase.

Widespread/concurrent MSK pain is very common among elderly people, affecting every second or third elderly person.

## Discussion

### Summary of evidence

In this review a great variation in prevalence of MSK disorders in older people were found. The most likely reasons for these differences are: 1) different pain definitions, 2) different prevalence periods, 3) different age intervals, and 4) the prevalence estimates were either divided by gender or only reported as a total prevalence estimate. Thus, it is impossible to determine any overall estimates on the prevalence of MSK problems in the elderly population.

Nevertheless, some general observations can be drawn from this review that needs to be discussed. Musculoskeletal disorders remain prevalent in the elderly population. Especially, OA is very common among elderly people, followed by knee pain, BP, and for women also OP. Pain mechanisms in the older population are poorly understood, but it is generally believed that pain at younger ages continues in the older ages [101]. Thus, pain in the elderly should be regarded as a continuum of pain from earlier years [101].

Women tend to report MSK pain significantly more often than men in almost all studies. This gender difference in pain reporting is well known, but the reason for this is probably multifactorial with both biological and psychosocial underlying mechanisms. These different pain mechanisms are beyond the scope of this paper to discuss in detail, but are presented in a review by Fillinghim et al. [102].

There is a general trend that prevalence estimates either remain fairly constant with increasing age or that they drop slightly in the oldest people, typically from 80 years of age and onwards. An exception from this is OP, where a steady increase is reported with increasing age.

There are several potential explanations for this decline in pain reporting with age. It may simply be a general birth cohort effect which may reflect both cultural and public health related differences between for example 40 year olds and 80 year olds [103]. This potential cohort effect may be more pronounced in cross-sectional studies, which were the only included studies in this review. A parallel to this may be that pain is accepted by the elderly as part of becoming old [104]. In other words, pain becomes a natural part of their life and therefore become less disturbing or simply ignored. It is also known that pressure pain decreases with age [105]. Finally, a decline in pain prevalences in the oldest old could be explained by a “survival of the fittest” phenomenon [103]. However, MSK pain itself does not lead to premature mortality per se [106-108]. Furthermore, this “biological elite” phenomenon is probably slowly diminishing as health and living standards in the World is generally improving and thus, more people are living longer and generally at better health

Finally, there is a considerable degree of overlapping MSK symptoms as approximately every second or third elderly have widespread MSK pain. This trend is most likely part of a continuum from widespread pain at younger ages as previously mentioned [101].

### Comparisons with other reviews

To our knowledge, no previous systematic literature reviews on a broader range of MSK conditions in elderly populations exist. However, a few reviews on some of our MSK conditions in the elderly populations were identified. Woolf and Pfleger reported high prevalence estimates in the elderly people for OA, RA, OP, and LBP in the developed countries [4]. In all four MSK diseases, the same age related increase in prevalence was found in their review, except for LBP where it remained fairly constant.

A literature review on LBP before 2000 found only 12 prevalence estimates specifically on elderly populations, but the authors were unable to make any general estimates mainly because of the different (or lack of) LBP definitions as well as the varying age intervals [109]. In a more recent LBP review published in 2006 on age related changes, concluded that “benign” LBP decreased with age, but that more severe LBP increased with age [110]. Due to the heterogeneity of these studies and the aim of their review, no attempt was made to provide any general LBP prevalence estimates.

Luime et al. published a review in 2004 on shoulder pain [111]. The point prevalence on subjects <70 ranging 7%-27% was very similar for subjects older than 70 (12-26%), but this may be due to the varying pain definitions.

Dagenais et al. found a steady increase in hip OA with increasing age, ranging from 5% (60–64 year olds) to 14% (85+ year olds), and being more prevalent in women [112].

It is impossible to compare our results with the abovementioned reviews, as they too fail to provide pooled estimates due to the high degree of heterogeneity across the included studies. Nevertheless, a general increase in prevalence with age and a gender difference were reported in all reviews, which is in accordance with our own findings.

### Methodological issues

The heterogeneity of pain definitions is already a well known problem, but undoubtedly, researchers have many good reasons for why they use a specific and perhaps unique pain definition. Unfortunately, this makes it impossible to draw any general conclusions based on the currently available literature. However, it would be recommendable if authors would at least report one or two additional standardised measures, such as the questions from the standardised Nordic questionnaire on musculoskeletal pain [113]. Although, journals restrict the sizes of their papers by limiting the number of words or tables and hence, decreasing the amount of information available from the studies, it is becoming more and more common to have supplementary tables published via the publishing journal’s website. Such tables could include valuable information on gender specific and total prevalence estimates for future reviews to calculate pooled prevalence estimates.

It also needs mentioning that nearly twice as many prevalence estimates could have been obtained from 82 additional studies, if only authors had reported age specific estimates. So, just like the standardisation of pain definitions is warranted, standardisation of age interval reporting would also be preferable. This way, more information on age related changes from the current literature could easily have been obtained.

In this review, we found that many authors state that their results are representative of the general population. However, only few actually document this. While many do their best at obtaining a random and representative target sample from the background population, an actual non-response analysis is rarely performed. For this reason, the risk of bias of the majority of the studies (65%) was deemed unclear. Studies were generally judged as having an “unclear” risk of bias because information was missing in the study description. In other words, the external validity of these studies is questionable, which is essential in epidemiological studies. It is therefore important to either report and/or adjust for non-response bias in future studies.

### Strengths and limitations of this review

Just like our included studies, our review has also some limitations that need to be addressed. We only included one electronic database (Pubmed) and thus, may have missed some relevant articles. Based on other reviews on similar MSK conditions, who have included other electronic databases (i.e. EMBASE, CINAHL, etc.), we may have missed between zero and 12% potentially relevant articles [109-112]. However, given the large heterogeneity and therefore lack of proper summary prevalence estimates, we doubt any missed articles would have had any major impact on our results. Our search strategy was also limited to the elderly population through MeSH terms. This may have lead to exclusion of some studies if for some reason they were not properly indexed in Pubmed. As only English language articles were included, any articles published in national non-English medical journals are missing in our literature review. Finally, the selection of articles was only conducted by one author, thus, there is a risk of missing potentially relevant articles. According to Edwards et al., an average of 9% of relevant articles may be missed (ranging between 0 and 32%) [114]. Thus, on average we may have missed approximately 8 articles.

The results from the included epidemiological studies must be viewed in light of the quality of these studies which depends on both the internal validity and if the results can be extrapolated to the background population (i.e. the external validity). In this review, the risk of bias rather than the quality of the studies were used as we wished to determine if the results were “believable” and not just if the “reporting” was satisfactory. The risk of bias assessment on randomised clinical trials is also recommended by the Cochrane Collaboration [8] and recently a set of risk of bias items were developed by Viswanathan et al. [9] which allowed us to design an assessment sheet well suited for our needs. However, assessing the risk of bias demands a high degree of judgement, is more time consuming, and may result in greater variability of interpretations of the studies [9,115]. Therefore, no attempt at adjusting the prevalence estimates based on the risk of bias judgment was made. Instead, we leave it up to the readers to decide on how to utilise our risk of bias judgments.

Because MSK pain may be reported as part of a larger health related publication and because a wide set of MSK conditions were included in our review, it was necessary to have rather broad search strategy. This in turn, resulted in a very large number of hits that had to be perused to seek for any potentially relevant articles. While the search may have been fairly sensitive in catching relevant articles it cannot be considered to be very specific. This becomes clear as less than 4% of the initial search results were retrieved and only 46% of those included. We did not attempt to specify the literature search any further as some of the included articles would have been missed, especially those articles where the reporting of MSK conditions are “secondary” findings.

Another limitation is the choice of only investigating the prevalence of MSK disorders among elderly people and, hence, excluding information on burden and cost-of-illness of these MSK conditions. Clearly, the presence of pain does not reflect how MSK problems affect older people on a daily basis. However, in the 2003 WHO report, Woolf and Pfleger reported that MSK conditions have a major societal impact in terms of reduced work disability, which would affect the “younger” elderly people aged 60–65, and result in an increased use of health care services [4]. Finally, with increasing OP, there is a high risk of fracture incidences. As most MSK conditions remain fairly common in the elderly populations and as the number of elderly people increases in the future, the socioeconomic burden of MSK in the elderly population will also increase. Thus, there will be a further need for health care professionals to deal with chronic MSK conditions among the elderly people.

### Future perspectives

This review has looked at the prevalence of a series of musculoskeletal conditions in the elderly population and will serve not only as a reference for future studies, but also as a guide for clinicians in general. Firstly, a larger population of geriatric patients must be expected in the future and thus calls for more attention on developing optimal geriatric patient management protocols. Secondly, it is important for a person to maintain a sufficient functional capacity in order to maintain an active life at older age [3]. In other words, political programmes as well as primary and secondary health care programmes accommodated to the future needs are necessary in order to maintain (or ideally improve) the quality of life in the elderly population.

## Conclusions

No overall estimate on the prevalence of MSK problems in the elderly population can be determined due to the heterogeneity of the studies. However, MSK disorders are common in the elderly population and women have more often MSK problems than men. There is a general trend that prevalence estimates either remain fairly constant or increase slightly with increasing age. However, for many MSK conditions, there is a slight decrease among the oldest (80+) people. Finally, many elderly people report multiple MSK pain sites.

## Competing interests

The authors declare that they have no competing interests.

## Authors’ contributions

RF planned the design of the study, conducted the literature search and wrote the initial draft of the manuscript. AR cross checked the extracted data including the risk of bias assessments. Both authors participated in writing the final manuscript.

## Supplementary Material

Additional file 1 **List of developed countries included in this literature review.** Included countries in this review based on advanced economies according to the International Monetary Foundation. Click here for file

Additional file 2 Search strategy – Pubmed.org. Click here for file

Additional file 3 **Overview of excluded articles.** All retrieved articles that were initially considered of relevance, but subsequently excluded because inclusion/exclusion criteria were not fulfilled. Click here for file

Additional file 4 **Risk of bias for all included studies.** All included studies were assessed for potential risk of bias. Click here for file
