# Supplementary material for: What is the prevalence of musculoskeletal problems in the elderly population in developed countries? A systematic critical literature review
Source: Chiropr Man Therap. 2012 Sep 24;20:31. doi: 10.1186/2045-709X-20-31 (PMC3507809; doi:10.1186/2045-709X-20-31)
Supplement: Additional file 1 — List of developed countries included in this literature review. Included countries in this review based on advanced economies according to the International Monetary Foundation. [file 2045-709X-20-31-S1.doc]

| **Additional file 1.** Included countries in this review based on advanced economies according to the International Monetary Foundation (IMF)*, in alphabetical order. | | |
| --- | --- | --- |
| Australia | Hong Kong | San Marino |
| Austria | Iceland | Singapore |
| Belgium | Ireland | Slovakia |
| Canada | Israel | Slovenia |
| Cyprus | Italy | South Korea |
| Czech Republic | Japan | Spain |
| Denmark | Luxembourg | Sweden |
| Estonia | Malta | Switzerland |
| Finland | Netherlands | Taiwan |
| France | New Zealand | United Kingdom |
| Germany | Norway | United States |
| Greece | Portugal |  |
| *www.imf.org | | |
