# Supplementary material for: What is the prevalence of musculoskeletal problems in the elderly population in developed countries? A systematic critical literature review
Source: Chiropr Man Therap. 2012 Sep 24;20:31. doi: 10.1186/2045-709X-20-31 (PMC3507809; doi:10.1186/2045-709X-20-31)
Supplement: Additional file 2 — Search strategy – Pubmed.org. [file 2045-709X-20-31-S2.doc]

**Additional file 2. Search strategy, Pubmed (**[**www.pubmed.org**](http://www.pubmed.org/)**).**

(("Musculoskeletal Diseases/epidemiology"[MeSH] OR “musculoskeletal”[All Fields])

OR

("Arthritis, Rheumatoid/epidemiology"[MeSH] OR "rheumatoid arthritis"[All Fields])

OR

("Osteoarthritis/epidemiology"[MeSH] OR "Osteoarthritis"[All Fields])

OR

("Osteoporosis/epidemiology"[MeSH] OR "Osteoporosis"[All Fields])

OR

("Neck Pain/epidemiology"[MeSH] OR "Back Pain/epidemiology"[MeSH] OR "Low Back Pain/epidemiology"[MeSH]))

AND

("Prevalence"[MeSH] OR "Incidence"[MeSH] OR "Cross-Sectional Studies"[MeSH] OR "Longitudinal Studies/statistics and numerical data"[MeSH])

AND

("humans"[MeSH] AND (Review[ptyp] OR Government Publications[ptyp] OR Journal Article[ptyp] OR Technical Report[ptyp])

AND

English[lang]

AND

("aged"[MeSH] OR "aged, 80 and over"[MeSH] OR “elderly”[All Fields])

AND

("2000/01/01"[PDAT] : "2011/07/01"[PDAT]))
