# Supplementary material for: What is the prevalence of musculoskeletal problems in the elderly population in developed countries? A systematic critical literature review
Source: Chiropr Man Therap. 2012 Sep 24;20:31. doi: 10.1186/2045-709X-20-31 (PMC3507809; doi:10.1186/2045-709X-20-31)
Supplement: Additional file 3 — Overview of excluded articles. All retrieved articles that were initially considered of relevance, but subsequently excluded because inclusion/exclusion criteria were not fulfilled. [file 2045-709X-20-31-S3.doc]

| **Additional file 3. Overview of excluded articles.** | | |
| --- | --- | --- |
| **Reference** |  | **Main reason for exclusion** |
| Ahlborg HG, Rosengren BE, Järvinen TL, Rogmark C, Nilsson JA, Sernbo I, Karlsson MK. Prevalence of osteoporosis and incidence of hip fracture in women--secular trends over 30 years. BMC Musculoskelet Disord. 2010 Mar 11;11:48 |  | No specific prevalence estimates on elderly aged 60 and over. |
| Abell JE, Hootman JM, Helmick CG. Prevalence and impact of arthritis among nursing home residents. Ann Rheum Dis. 2004 May;63(5):591-4. |  | No specific MSK condition reported on elderly aged 60 and over. |
| Ahacic K, Kåreholt I. Prevalence of musculoskeletal pain in the general Swedish population from 1968 to 2002: age, period, and cohort patterns. Pain. 2010 Oct;151(1):206-14. Epub 2010 Aug 13. |  | No specific MSK condition reported on elderly aged 60 and over. |
| Badley EM, Ansari H. Arthritis and arthritis-attributable activity limitations in the United States and Canada: a cross-border comparison. Arthritis Care Res (Hoboken). 2010 Mar;62(3):308-15. |  | No specific MSK condition reported on elderly aged 60 and over. |
| Bedson J, Jordan K, Croft P. The prevalence and history of knee osteoarthritis in general practice: a case-control study. Fam Pract. 2005 Feb;22(1):103-8. Epub 2005 Jan 7 |  | No actual population-based prevalence estimates provided on elderly aged 60 and over. |
| Bergman S, Herrström P, Högström K, Petersson IF, Svensson B, Jacobsson LT. Chronic musculoskeletal pain, prevalence rates, and sociodemographic associations in a Swedish population study. J Rheumatol. 2001 Jun;28(6):1369-77 |  | No specific prevalence estimates on elderly aged 60 and over. |
| Bingefors K, Isacson D. Epidemiology, co-morbidity, and impact on health-related quality of life of self-reported headache and musculoskeletal pain--a gender perspective. Eur J Pain. 2004 Oct;8(5):435-50. |  | No specific prevalence estimates on elderly aged 60 and over. |
| Björck-van Dijken C, Fjellman-Wiklund A, Hildingsson C. Low back pain, lifestyle factors and physical activity: a population based-study. J Rehabil Med. 2008 Nov;40(10):864-9. |  | No actual population-based prevalence estimates provided on elderly aged 60 and over. |
| Blyth FM, March LM, Cousins MJ. Chronic pain-related disability and use of analgesia and health services in a Sydney community. Med J Aust. 2003 Jul 21;179(2):84-7 |  | No specific prevalence estimates on elderly aged 60 and over. |
| Bolen J, Schieb L, Hootman JM, Helmick CG, Theis K, Murphy LB, Langmaid G. Differences in the prevalence and severity of arthritis among racial/ethnic groups in the United States, National Health Interview Survey, 2002, 2003, and 2006. Prev Chronic Dis. 2010 May;7(3):A64. Epub 2010 Apr 15. |  | No specific MSK condition reported on elderly aged 60 and over. |
| Bot SD, van der Waal JM, Terwee CB, van der Windt DA, Schellevis FG, Bouter LM, Dekker J. Incidence and prevalence of complaints of the neck and upper extremity in general practice. Ann Rheum Dis. 2005 Jan;64(1):118-23 |  | No specific prevalence estimates on elderly aged 60 and over. |
| Bridges SL Jr, Causey ZL, Burgos PI, Huynh BQ, Hughes LB, Danila MI, van Everdingen A, Ledbetter S, Conn DL, Tamhane A, Westfall AO, Jonas BL, Callahan LF, Smith EA, Brasington R, Moreland LW, Alarcón GS, van der Heijde DM. Radiographic severity of rheumatoid arthritis in African Americans: results from a multicenter observational study. Arthritis Care Res (Hoboken). 2010 May;62(5):624-31. |  | No specific prevalence estimates on elderly aged 60 and over. |
| Busija L, Hollingsworth B, Buchbinder R, Osborne RH. Role of age, sex, and obesity in the higher prevalence of arthritis among lower socioeconomic groups: a population-based survey. Arthritis Rheum. 2007 May 15;57(4):553-61. |  | No specific MSK condition reported on elderly aged 60 and over. |
| Carbonell J, Cobo T, Balsa A, Descalzo MA, Carmona L; SERAP Study Group. The incidence of rheumatoid arthritis in Spain: results from a nationwide primary care registry. Rheumatology (Oxford). 2008 Jul;47(7):1088-92. Epub 2008 May 29 |  | No specific prevalence estimates on elderly aged 60 and over. |

| **Table 3 (cont’d).** Overview of excluded articles. | | |
| --- | --- | --- |
| **Reference** |  | **Main reason for exclusion** |
| Carmona L, Villaverde V, Hernández-García C, Ballina J, Gabriel R, Laffon A; EPISER Study Group. The prevalence of rheumatoid arthritis in the general population of Spain. Rheumatology (Oxford). 2002 Jan;41(1):88-95 |  | Results included in another article (PMID 11602475) |
| Centers for Disease Control and Prevention (CDC). Prevalence of doctor-diagnosed arthritis and arthritis-attributable activity limitation --- United States, 2007-2009. MMWR Morb Mortal Wkly Rep. 2010 Oct 8;59(39):1261-5. |  | No specific MSK condition reported on elderly aged 60 and over. |
| Centers for Disease Control and Prevention (CDC). Prevalence of doctor-diagnosed arthritis and arthritis-attributable activity limitation—United States, 2003-2005. MMWR Morb Mortal Wkly Rep. 2006 Oct 13;55(40):1089-92. Erratum in: MMWR Morb Mortal Wkly Rep. 2007 Jan 26;56(3):55. MMWR Morb Mortal Wkly Rep. 2006 Oct 20;55(41):1129 |  | No specific MSK condition reported on elderly aged 60 and over. |
| Centers for Disease Control and Prevention (CDC). Prevalence of doctor-diagnosed arthritis and arthritis-attributable effects among Hispanic adults, by Hispanic subgroup--United States, 2002, 2003, 2006, and 2009. MMWR Morb Mortal Wkly Rep. 2011 Feb 18;60(6):167-71 |  | No specific MSK condition reported on elderly aged 60 and over. |
| Centers for Disease Control and Prevention (CDC). Prevalence of self-reported arthritis or chronic joint symptoms among adults--United States, 2001. MMWR Morb Mortal Wkly Rep. 2002 Oct 25;51(42):948-50. |  | No specific MSK condition reported on elderly aged 60 and over. |
| Centers for Disease Control and Prevention (CDC). Public health and aging: projected prevalence of self-reported arthritis or chronic joint symptoms among persons aged >65 years--United States, 2005-2030. MMWR Morb Mortal Wkly Rep. 2003 May 30;52(21):489-91. |  | No specific MSK condition reported on elderly aged 60 and over. |
| Cho NH, Kim S, Kwon DJ, Kim HA. The prevalence of hallux valgus and its association with foot pain and function in a rural Korean community. J Bone Joint Surg Br. 2009 Apr;91(4):494-8. |  | No specific prevalence estimates on elderly aged 60 and over. |
| Cook C, Pietrobon R, Hegedus E. Osteoarthritis and the impact on quality of life health indicators. Rheumatol Int. 2007 Feb;27(4):315-21. Epub 2006 Nov 15. |  | No specific prevalence estimates on elderly aged 60 and over. |
| Croft PR, Lewis M, Papageorgiou AC, Thomas E, Jayson MI, Macfarlane GJ, Silman AJ. Risk factors for neck pain: a longitudinal study in the general population. Pain. 2001 Sep;93(3):317-25. |  | No specific prevalence estimates on elderly aged 60 and over. |
| Dahaghin S, Bierma-Zeinstra SM, Ginai AZ, Pols HA, Hazes JM, Koes BW. Prevalence and pattern of radiographic hand osteoarthritis and association with pain and disability (the Rotterdam study). Ann Rheum Dis. 2005 May;64(5):682-7. Epub 2004 Sep 16 |  | No specific prevalence estimates on elderly aged 60 and over. |
| Dahaghin S, Bierma-Zeinstra SM, Reijman M, Pols HA, Hazes JM, Koes BW. Prevalence and determinants of one month hand pain and hand related disability in the elderly (Rotterdam study). Ann Rheum Dis. 2005 Jan;64(1):99-104. Erratum in: Ann Rheum Dis. 2005 Mar;64(3):516. |  | No specific prevalence estimates on elderly aged 60 and over. |
| Damborg F, Engell V, Andersen M, Kyvik KO, Thomsen K. Prevalence, concordance, and heritability of Scheuermann kyphosis based on a study of twins. J Bone Joint Surg Am. 2006 Oct;88(10):2133-6. |  | No specific prevalence estimates on elderly aged 60 and over. |
| D'Astolfo CJ, Humphreys BK. A record review of reported musculoskeletal pain in an Ontario long term care facility. BMC Geriatr. 2006 Mar 23;6:5. |  | No specific prevalence estimates on elderly aged 60 and over. |
| Di Iorio A, Abate M, Guralnik JM, Bandinelli S, Cecchi F, Cherubini A, Corsonello A, Foschini N, Guglielmi M, Lauretani F, Volpato S, Abate G, Ferrucci L. From chronic low back pain to disability, a multifactorial mediated pathway: the InCHIANTI study. Spine (Phila Pa 1976). 2007 Dec 15;32(26):E809-15 |  | Results included in another article (PMID 16648752) |

| **Table 3 (cont’d).** Overview of excluded articles. | | |
| --- | --- | --- |
| **Reference** |  | **Main reason for exclusion** |
| Donald IP, Foy C. A longitudinal study of joint pain in older people. Rheumatology (Oxford). 2004 Oct;43(10):1256-60. Epub 2004 Jul 13. |  | No specific MSK condition reported on elderly aged 60 and over. |
| Dufour AB, Broe KE, Nguyen US, Gagnon DR, Hillstrom HJ, Walker AH, Kivell E, Hannan MT. Foot pain: is current or past shoewear a factor? Arthritis Rheum. 2009 Oct 15;61(10):1352-8 |  | No specific prevalence estimates on elderly aged 60 and over. |
| Dunlop DD, Manheim LM, Song J, Chang RW. Arthritis prevalence and activity limitations in older adults. Arthritis Rheum. 2001 Jan;44(1):212-21. |  | No specific MSK condition reported on elderly aged 60 and over. |
| Feinglass J, Nelson C, Lawther T, Chang RW. Chronic joint symptoms and prior arthritis diagnosis in community surveys: implications for arthritis prevalence estimates. Public Health Rep. 2003 May-Jun;118(3):230-9 |  | No specific MSK condition reported on elderly aged 60 and over. |
| Fleming DM, Cross KW, Barley MA. Recent changes in the prevalence of diseases presenting for health care. Br J Gen Pract. 2005 Aug;55(517):589-95 |  | No specific prevalence estimates on elderly aged 60 and over. |
| Golightly YM, Hannan MT, Shi XA, Helmick CG, Renner JB, Jordan JM. Association of foot symptoms with self-reported and performance-based measures of physical function: The Johnston County osteoarthritis project. Arthritis Care Res (Hoboken). 2011 May;63(5):654-9. doi: 10.1002/acr.20432. |  | No specific prevalence estimates on elderly aged 60 and over. |
| Gross DP, Ferrari R, Russell AS, Battié MC, Schopflocher D, Hu RW, Waddell G, Buchbinder R. A population-based survey of back pain beliefs in Canada. Spine (Phila Pa 1976). 2006 Aug 15;31(18):2142-5 |  | No specific prevalence estimates on elderly aged 60 and over. |
| Gummesson C, Isacsson SO, Isacsson AH, Andersson HI, Ektor-Andersen J, Ostergren PO, Hanson B; Malmö Shoulder-Neck Study group. The transition of reported pain in different body regions--a one-year follow-up study. BMC Musculoskelet Disord. 2006 Feb 23;7:17 |  | No specific prevalence estimates on elderly aged 60 and over. |
| Hartvigsen J, Christensen K, Frederiksen H. Back pain remains a common symptom in old age. a population-based study of 4486 Danish twins aged 70-102. Eur Spine J. 2003 Oct;12(5):528-34. Epub 2003 May 14. |  | Results included in another article (PMID 12748896) |
| Hasselström J, Liu-Palmgren J, Rasjö-Wrååk G. Prevalence of pain in general practice. Eur J Pain. 2002;6(5):375-85. |  | Unable to extract or determine prevalence estimates on elderly aged 60 and over. |
| Hill J, Lewis M, Papageorgiou AC, Dziedzic K, Croft P. Predicting persistent neck pain: a 1-year follow-up of a population cohort. Spine (Phila Pa 1976). 2004 Aug 1;29(15):1648-54 |  | No specific prevalence estimates on elderly aged 60 and over. |
| Hirsch R, Guralnik JM, Ling SM, Fried LP, Hochberg MC. The patterns and prevalence of hand osteoarthritis in a population of disabled older women: The Women's Health and Aging Study. Osteoarthritis Cartilage. 2000;8 Suppl A:S16-21 |  | Not a representative sample of the target population. |
| Hong JY, Suh SW, Modi HN, Hur CY, Song HR, Park JH. The prevalence and radiological findings in 1347 elderly patients with scoliosis. J Bone Joint Surg Br. 2010 Jul;92(7):980-3 |  | Not a representative sample of the target population. |
| Hootman JM, Helmick CG. Projections of US prevalence of arthritis and associated activity limitations. Arthritis Rheum. 2006 Jan;54(1):226-9. |  | No specific MSK condition reported on elderly aged 60 and over. |
| Horikawa K, Kasai Y, Yamakawa T, Sudo A, Uchida A. Prevalence of osteoarthritis, osteoporotic vertebral fractures, and spondylolisthesis among the elderly in a Japanese village. J Orthop Surg (Hong Kong). 2006 Apr;14(1):9-12. |  | Unable to extract or determine prevalence estimates on elderly aged 60 and over. |
| Hsu FC, Starkebaum G, Boyko EJ, Dominitz JA. Prevalence of rheumatoid arthritis and hepatitis C in those age 60 and older in a US population based study. J Rheumatol. 2003 Mar;30(3):455-8. |  | Not a representative sample of the target population. |

| **Table 3 (cont’d).** Overview of excluded articles. | | |
| --- | --- | --- |
| **Reference** |  | **Main reason for exclusion** |
| Huisstede BM, Wijnhoven HA, Bierma-Zeinstra SM, Koes BW, Verhaar JA, Picavet S. Prevalence and characteristics of complaints of the arm, neck, and/or shoulder (CANS) in the open population. Clin J Pain. 2008 Mar-Apr;24(3):253-9. |  | No specific MSK condition reported on elderly aged 60 and over. |
| Hüppe A, Müller K, Raspe H. Is the occurrence of back pain in Germany decreasing? Two regional postal surveys a decade apart. Eur J Public Health. 2007 Jun;17(3):318-22. Epub 2006 Sep 23 |  | No specific prevalence estimates on elderly aged 60 and over. |
| Häussler B, Gothe H, Göl D, Glaeske G, Pientka L, Felsenberg D. Epidemiology, treatment and costs of osteoporosis in Germany--the BoneEVA Study. Osteoporos Int. 2007 Jan;18(1):77-84. Epub 2006 Sep 19 |  | No actual population-based prevalence estimates provided on elderly aged 60 and over. |
| Haara MM, Manninen P, Kroger H, Arokoski JP, Karkkainen A, Knekt P, Aromaa A, Heliovaara M. Osteoarthritis of finger joints in Finns aged 30 or over: prevalence, determinants, and association with mortality. Ann Rheum Dis. 2003 Feb;62(2):151-8. |  | Too specific MSK conditions (single finger joints OA). |
| Ihlebaek C, Eriksen HR, Ursin H. Prevalence of subjective health complaints (SHC) in Norway. Scand J Public Health. 2002;30(1):20-9. |  | No specific prevalence estimates on elderly aged 60 and over. |
| Ingvarsson T. Prevalence and inheritance of hip osteoarthritis in Iceland. Acta Orthop Scand Suppl. 2000 Dec;298:1-46 |  | Not a representative sample of the target population. |
| Jiménez-Sánchez S, Jiménez-García R, Hernández-Barrera V, Villanueva-Martínez M, Ríos-Luna A, Fernández-de-las-Peñas C. Has the prevalence of invalidating musculoskeletal pain changed over the last 15 years (1993-2006)? A Spanish population-based survey. J Pain. 2010 Jul;11(7):612-20. Epub 2010 Mar 31. |  | No specific MSK condition reported on elderly aged 60 and over. |
| Johannes CB, Le TK, Zhou X, Johnston JA, Dworkin RH. The prevalence of chronic pain in United States adults: results of an Internet-based survey. J Pain. 2010 Nov;11(11):1230-9. |  | No specific prevalence estimates on elderly aged 60 and over. |
| Kaipiainen-Seppänen O, Aho K, Nikkarinen M. Regional differences in the incidence of rheumatoid arthritis in Finland in 1995. Ann Rheum Dis. 2001 Feb;60(2):128-32 |  | No specific prevalence estimates on elderly aged 60 and over. |
| Kalichman L, Cole R, Kim DH, Li L, Suri P, Guermazi A, Hunter DJ. Spinal stenosis prevalence and association with symptoms: the Framingham Study. Spine J. 2009 Jul;9(7):545-50. |  | No specific MSK condition reported on elderly aged 60 and over. |
| Kalichman L, Li L, Kim DH, Guermazi A, Berkin V, O'Donnell CJ, Hoffmann U, Cole R, Hunter DJ. Facet joint osteoarthritis and low back pain in the community-based population. Spine (Phila Pa 1976). 2008 Nov 1;33(23):2560-5. |  | No specific MSK condition reported on elderly aged 60 and over. |
| Kebaish KM, Neubauer PR, Voros GD, Khoshnevisan MA, Skolasky RL. Scoliosis in adults aged forty years and older: prevalence and relationship to age, race, and gender. Spine (Phila Pa 1976). 2011 Apr 20;36(9):731-6. |  | Not a representative sample of the target population. |
| Kirk JK, Spangler JG, Celestino FS. Prevalence of osteoporosis risk factors and treatment among women aged 50 years and older. Pharmacotherapy. 2000 Apr;20(4):405-9. |  | No specific prevalence estimates on elderly aged 60 and over. |
| Knox SA, Harrison CM, Britt HC, Henderson JV. Estimating prevalence of common chronic morbidities in Australia. Med J Aust. 2008 Jul 21;189(2):66-70. |  | No specific prevalence estimates on elderly aged 60 and over. |
| Kopec JA, Rahman MM, Berthelot JM, Le Petit C, Aghajanian J, Sayre EC, Cibere J, Anis AH, Badley EM. Descriptive epidemiology of osteoarthritis in British Columbia, Canada. J Rheumatol. 2007 Feb;34(2):386-93. |  | No site-specific MSK condition reported on elderly aged 60 and over. |
| Kramer PA. Prevalence and distribution of spinal osteoarthritis in women. Spine (Phila Pa 1976). 2006 Nov 15;31(24):2843-8. |  | Not a representative sample of the target population. |

| **Table 3 (cont’d).** Overview of excluded articles. | | |
| --- | --- | --- |
| **Reference** |  | **Main reason for exclusion** |
| Leveille SG, Fried L, Guralnik JM. Disabling symptoms: what do older women report?. J Gen Intern Med. 2002 ct;17(10):766-73. |  | No specific MSK condition reported on elderly aged 60 and over. |
| Linsell L, Dawson J, Zondervan K, Rose P, Carr A, Randall T, Fitzpatrick R. Population survey comparing older adults with hip versus knee pain in primary care. Br J Gen Pract. 2005 Mar;55(512):192-8. |  | Results included in another article (PMID 14762225) |
| Lohmander LS, Gerhardsson de Verdier M, Rollof J, Nilsson PM, Engström G. Incidence of severe knee and hip osteoarthritis in relation to different measures of body mass: a population-based prospective cohort study. Ann Rheum Dis. 2009 Apr;68(4):490-6. Epub 2008 May 8 |  | No specific prevalence estimates on elderly aged 60 and over. |
| Makris UE, Fraenkel L, Han L, Leo-Summers L, Gill TM. Epidemiology of restricting back pain in community-living older persons. J Am Geriatr Soc. 2011 Apr;59(4):610-4 |  | No specific prevalence estimates on elderly aged 60 and over. |
| Mannoni A, Briganti MP, Di Bari M, Ferrucci L, Serni U, Masotti G, Marchionni N. Prevalence of symptomatic hand osteoarthritis in community-dwelling older persons: the ICARe Dicomano study. Insufficienza Cardiaca negli Anzizni Residenti a Dicomano. Osteoarthritis Cartilage. 2000;8 Suppl A:S11-3 |  | Results included in another article (which contains more results) (PMID 11156485). |
| Mili F, Helmick CG, Zack MM. Prevalence of arthritis: analysis of data from the US Behavioral Risk Factor Surveillance System, 1996-99. J Rheumatol. 2002 Sep;29(9):1981-8 |  | No specific MSK condition reported on elderly aged 60 and over. |
| Myasoedova E, Crowson CS, Kremers HM, Therneau TM, Gabriel SE. Is the incidence of rheumatoid arthritis rising?: results from Olmsted County, Minnesota, 1955-2007. Arthritis Rheum. 2010 Jun;62(6):1576-82 |  | No specific prevalence estimates on elderly aged 60 and over. |
| Natvig B, Bruusgaard D, Eriksen W. Localized low back pain and low back pain as part of widespread musculoskeletal pain: two different disorders? A cross-sectional population study. J Rehabil Med. 2001 Jan;33(1):21-5. |  | No specific prevalence estimates on elderly aged 60 and over. |
| Niu J, Zhang Y, LaValley M, Chaisson CE, Aliabadi P, Felson DT. Symmetry and clustering of symptomatic hand osteoarthritis in elderly men and women: the Framingham Study. Rheumatology (Oxford). 2003 Feb;42(2):343-8. |  | No specific prevalence estimates on elderly aged 60 and over. |
| Parkinson L, Gibson R, Robinson I, Byles J. Older women and arthritis: tracking impact over time. Australas J Ageing. 2010 Dec;29(4):155-60. |  | No specific MSK condition reported on elderly aged 60 and over. |
| Pérez-Castrillón JL, Martín-Escudero JC, del Pino-Montes J, Blanco FS, Martín FJ, Paredes MG, Fernández FP, Arés TA. Prevalence of osteoporosis using DXA bone mineral density measurements at the calcaneus: cut-off points of diagnosis and exclusion of osteoporosis. J Clin Densitom. 2005 Winter;8(4):404-8. |  | Testing different T-score cut-off points of calcaneus OP. |
| Perruccio AV, Badley EM. Proxy reporting and the increasing prevalence of arthritis in Canada. Can J Public Health. 2004 May-Jun;95(3):169-73. |  | No specific MSK condition reported on elderly aged 60 and over. |
| Perruccio AV, Power JD, Badley EM. Revisiting arthritis prevalence projections--it's more than just the aging of the population. J Rheumatol. 2006 Sep;33(9):1856-62 |  | No specific MSK condition reported on elderly aged 60 and over. |
| Persson G, Barlow L, Karlsson A, Rosén M, Stefansson CG, Theorell T, Tüll P, Aberg A. Chapter 3. Major health problems. Health in Sweden: The National Public Health Report 2001. Scand J Public Health Suppl. 2001;58:37-102 |  | A summary/review (a short background introduction on disorders of the locomotive system in Sweden). |

| **Table 3 (cont’d).** Overview of excluded articles. | | |
| --- | --- | --- |
| **Reference** |  | **Main reason for exclusion** |
| Plesh O, Adams SH, Gansky SA. Racial/Ethnic and gender prevalences in reported common pains in a national sample. J Orofac Pain. 2011 Winter;25(1):25-31. |  | No general prevalence estimates of the whole study sample on elderly aged 60 and over (data divided by racial/ethnicity). |
| Puts MT, Deeg DJ, Hoeymans N, Nusselder WJ, Schellevis FG. Changes in the prevalence of chronic disease and the association with disability in the older Dutch population between 1987 and 2001. Age Ageing. 2008 Mar;37(2):187-93. Epub 2008 Feb 4. |  | No specific prevalence estimates on elderly aged 60 and over. |
| Raspe H, Matthis C, Croft P, O'Neill T; European Vertebral Osteoporosis Study Group. Variation in back pain between countries: the example of Britain and Germany. Spine (Phila Pa 1976). 2004 May 1;29(9):1017-21; |  | No specific prevalence estimates on elderly aged 60 and over. |
| Reichenbach S, Dieppe PA, Nüesch E, Williams S, Villiger PM, Jüni P. Association of bone attrition with knee pain, stiffness and disability: a cross-sectional study. Ann Rheum Dis. 2011 Feb;70(2):293-8. Epub 2010 Sep 24. |  | No specific prevalence estimates on elderly aged 60 and over. |
| Ritzwoller DP, Crounse L, Shetterly S, Rublee D. The association of comorbidities, utilization and costs for patients identified with low back pain. BMC Musculoskelet Disord. 2006 Sep 18;7:72. |  | No actual population-based prevalence estimates provided on elderly aged 60 and over. |
| Rodríguez LA, Tolosa LB, Ruigómez A, Johansson S, Wallander MA. Rheumatoid arthritis in UK primary care: incidence and prior morbidity. Scand J Rheumatol. 2009 May-Jun;38(3):173-7 |  | No specific prevalence estimates on elderly aged 60 and over. |
| Roux CH, Saraux A, Le Bihan E, Fardellone P, Guggenbuhl P, Fautrel B, Masson C, Chary-Valckenaere I, Cantagrel A, Juvin R, Flipo RM, Euller-Ziegler L, Coste J, Guillemin F. Rheumatoid arthritis and spondyloarthropathies: geographical variations in prevalence in France. J Rheumatol. 2007 Jan;34(1):117-22. Epub 2006 Nov 15. |  | No specific prevalence estimates on elderly aged 60 and over. |
| Rustøen T, Wahl AK, Hanestad BR, Lerdal A, Paul S, Miaskowski C. Age and the experience of chronic pain: differences in health and quality of life among younger, middle-aged, and older adults. Clin J Pain. 2005 Nov-Dec;21(6):513-23. |  | Not a representative sample of the target population. |
| Sallin U, Mellström D, Eggertsen R. Osteoporosis in a nursing home, determined by the DEXA technique. Med Sci Monit. 2005 Feb;11(2):CR67-70. |  | No specific prevalence estimates on elderly aged 60 and over. |
| Schneider S, Schmitt G, Richter W. Prevalence and correlates of inflammatory arthritis in Germany: data from the First National Health Survey. Rheumatol Int. 2006 Nov;27(1):29-38. Epub 2006 Jul 4 |  | No specific MSK condition reported on elderly aged 60 and over. |
| Simard JF, Mittleman MA. Prevalent rheumatoid arthritis and diabetes among NHANES III participants aged 60 and older. J Rheumatol. 2007 Mar;34(3):469-73. Epub 2007 Dec 15. |  | No actual population-based prevalence estimates on elderly aged 60 and over. |
| Sjøgren P, Ekholm O, Peuckmann V, Grønbaek M. Epidemiology of chronic pain in Denmark: an update. Eur J Pain. 2009 Mar;13(3):287-92. Epub 2008 Jun 10. |  | No specific prevalence estimates on elderly aged 60 and over. |
| Smith J, Inderjeeth C, Lewin G. Osteoporosis risk in a home care setting. Australas J Ageing. 2009 Jun;28(2):75-80 |  | No specific prevalence estimates on elderly aged 60 and over. |
| Sun J, Gooch K, Svenson LW, Bell NR, Frank C. Estimating osteoarthritis incidence from population-based administrative health care databases. Ann Epidemiol. 2007 Jan;17(1):51-6. Epub 2006 Oct 4 |  | No specific prevalence estimates on elderly aged 60 and over. |
| Söderlin MK, Börjesson O, Kautiainen H, Skogh T, Leirisalo-Repo M. Annual incidence of inflammatory joint diseases in a population based study in southern Sweden. Ann Rheum Dis. 2002 Oct;61(10):911-5. |  | No specific prevalence estimates on elderly aged 60 and over. |

| **Table 3 (cont’d).** Overview of excluded articles. | | |
| --- | --- | --- |
| **Reference** |  | **Main reason for exclusion** |
| Tenenhouse A, Joseph L, Kreiger N, Poliquin S, Murray TM, Blondeau L, Berger C, Hanley DA, Prior JC; CaMos Research Group.Canadian Multicentre Osteoporosis Study. Estimation of the prevalence of low bone density in Canadian women and men using a population-specific DXA reference standard: the Canadian Multicentre Osteoporosis Study (CaMos). Osteoporos Int. 2000;11(10):897-904. |  | Not a representative sample of the target population. |
| Toba N, Sakai A, Aoyagi K, Yoshida S, Honda S, Nakamura T. Prevalence and involvement patterns of radiographic hand osteoarthritis in Japanese women: the Hizen-Oshima Study. J Bone Miner Metab. 2006;24(4):344-8. |  | Too specific MSK conditions (single finger joints OA). |
| Tsuji T, Matsuyama Y, Sato K, Hasegawa Y, Yimin Y, Iwata H. Epidemiology of low back pain in the elderly: correlation with lumbar lordosis. J Orthop Sci. 2001;6(4):307-11 |  | Not a representative sample of the target population. |
| van den Bussche H, Koller D, Kolonko T, Hansen H, Wegscheider K, Glaeske G, von Leitner EC, Schäfer I, Schön G. Which chronic diseases and disease combinations are specific to multimorbidity in the elderly? Results of a claims data based cross-sectional study in Germany. BMC Public Health. 2011 Feb 14;11:101 |  | No specific MSK condition reported on elderly aged 60 and over. |
| Vradenburg JA, Simoes EJ, Jackson-Thompson J, Murayi T. The prevalence of arthritis and activity limitation and their predictors in Missouri. J Community Health. 2002 Apr;27(2):91-107 |  | No specific prevalence estimates on elderly aged 60 and over. |
| Walker BF, Muller R, Grant WD. Low back pain in Australian adults: prevalence and associated disability. J Manipulative Physiol Ther. 2004 May;27(4):238-44 |  | Too specific MSK conditions (graded sub groups of LBP). |
| Wang PP, Elsbett-Koeppen R, Geng G, Badley EM. Arthritis prevalence and place of birth: findings from the 1994 Canadian National Population Health Survey. Am J Epidemiol. 2000 Sep 1;152(5):442-5. |  | No specific MSK condition reported on elderly aged 60 and over. |
| Wijnhoven HA, de Vet HC, Picavet HS. Explaining sex differences in chronic musculoskeletal pain in a general population. Pain. 2006 Sep;124(1-2):158-66. Epub 2006 May 22. |  | No specific prevalence estimates on elderly aged 60 and over. |
| Wijnhoven HA, de Vet HC, Picavet HS. Prevalence of musculoskeletal disorders is systematically higher in women than in men. Clin J Pain. 2006 Oct;22(8):717-24 |  | No specific prevalence estimates on elderly aged 60 and over. |
| Wilder FV, Barrett JP, Farina EJ. Joint-specific prevalence of osteoarthritis of the hand. Osteoarthritis Cartilage. 2006 Sep;14(9):953-7. Epub 2006 Jun 8 |  | Too specific MSK conditions (single finger joints OA). |
| Wood LR, Peat G, Thomas E, Duncan R. Knee osteoarthritis in community-dwelling older adults: are there characteristic patterns of pain location? Osteoarthritis Cartilage. 2007 Jun;15(6):615-23. Epub 2007 Feb 1. |  | No specific MSK condition reported on elderly aged 60 and over. |
