# Supplementary material for: What is the prevalence of musculoskeletal problems in the elderly population in developed countries? A systematic critical literature review
Source: Chiropr Man Therap. 2012 Sep 24;20:31. doi: 10.1186/2045-709X-20-31 (PMC3507809; doi:10.1186/2045-709X-20-31)
Supplement: Additional file 4 — Risk of bias for all included studies. All included studies were assessed for potential risk of bias. [file 2045-709X-20-31-S4.doc]

**Additional file 4. Risk of bias for all included studies. For reference number please see reference list in manuscript.**

| **Author [reference]** | **Year** | **Inclusion/exclusion**  **criteria clearly stated** | **valid /reliable inclusion/**  **exclusion criteria** | **Valid and reliable**  **outcome measures** | **Level of detail of**  **the outcome** | **Overall risk of bias** | **Comments** |
| --- | --- | --- | --- | --- | --- | --- | --- |
| Andrianakos [10] | 2006 | + | + | **?** | + | **Low** | Low risk of recall bias despite efforts to use valid methods. Possible risk of a cohort effect given the long collection period; 1966-99. |
| Andrianakos [23] | 2006 | + | + | + | + | **Low** | Low risk of recall bias despite efforts to use valid methods. Possible risk of a cohort effect given the long collection period; 1966-99. |
| Andrianakos [41] | 2003 | + | + | + | + | **Low** | Low risk of recall bias despite efforts to use valid methods. Possible risk of a cohort effect given the long collection period; 1966-99. |
| Badlissi [95] | 2005 | **–** | **–** | **?** | + | **High** | High risk of selection and sampling bias.  Risk of information bias due to the mix of prevalence (1 wk and/or 1 mo). |
| Baek [59] | 2010 | **?** | **?** | **?** | **–** | **High** | Unclear if study sample is representative of target population.  Risk of information bias (combination of two different prevalence periods). |
| Biino [42] | 2011 | **?** | **?** | **?** | + | **Unclear** | Potential risk of sampling bias/non-response bias. |
| Bleicher [43] | 2010 | **?** | **?** | **?** | + | **Unclear** | Risk of sampling and non-response bias. |
| Carmona [11] | 2001 | + | **?** | + | + | **Low** | An actual non-response analysis has not been conducted, but responders are compared with the general population. |
| Cecchi [60] | 2006 | **?** | **?** | **?** | **?** | **Unclear** | Risk of information bias: The MSK definition is unclear and not validated. |
| Cheng [53] | 2009 | **?** | **?** | **?** | **?** | **Unclear** | High risk of reporting bias. |
| Chiu [61] | 2006 | **?** | **?** | **?** | **?** | **Unclear** | Risk of sampling and recall bias. Measurement bias may also be present. A non-response analysis has not been conducted. |
| Christmas [90] | 2002 | **?** | + | **?** | + | **Unclear** | The NHANES surveys have been shown to be representative of the target population and great efforts have been made to validate the results. However, the description/reporting in this article is limited. |
| Chung [116] | 2010 | **?** | **?** | + | + | **Unclear** | Unclear if study sample is representative of the target population. More details on cohort found in another (not referenced) article |
| Collerton [12] | 2009 | + | **?** | **?** | **–** | **Unclear** | Risk of reporting and information bias (GP records and questionnaire not validated). |
| Croft [93] | 2005 | **?** | **?** | + | + | **Unclear** | Unclear if responders are representative of the target population. A non-response analysis has not been conducted. |
| Cui [44] | 2008 | **?** | **?** | **?** | +  + | **Unclear** | Unclear if responders are representative of the target population. A non-response analysis has not been conducted. |
| Dawson [91] | 2004 | + | **?** | **?** | + | **Unclear** | A non-response analysis has not been conducted. |
| Denard [62] | 2010 | **?** | **?** | **?** | + | **Unclear** | Unclear if responders are representative of the target population. |
| Dillon [24] | 2007 | **?** | + | + | + | **Low** | NHANES III is well described elsewhere and shows a low risk of bias |
| Docking [63] | 2011 | **?** | **?** | **?** | **–** | **Unclear** | Unclear if study sample is representative of the target population. Definition of MSK condition is not stated or too ambiguous. |
| Dunn [99] | 2004 | **?** | **?** | **?** | + | **Unclear** | Unclear if responders are representative of the target population. A non-response analysis has not been conducted. |
| Englund [13] | 2010 | + | + | + | +  + | **Low** | Unclear how RA diagnosis was made, but since it had to be confirmed at minimum two occasions it seems valid. |
| Fernandez-Lopez [25] | 2008 | + | + | + | + | **Low** | Study well is described and indicates a low risk of bias |
| Freburger [64] | 2009 | **?** | **?** | + | + | **Unclear** | Unclear if responders are representative of the target population. |
| Goode [65] | 2010 | **?** | **?** | + | + | **Unclear** | Unclear if responders are representative of the target population. |
| Goubert [66] | 2004 | **–** | **?** | **?** | **–** | **Unclear** | Unclear if responders are representative of the target population. |
| Guez [67] | 2002 | **?** | **?** | **?** | **–** | **Unclear** | Unclear if responders are representative of the target population. |
| Gummesson [89] | 2003 | **?** | **?** | **?** | **?** | **Unclear** | Unclear if responders are representative of the target population. |
| Hanova [14] | 2006 | + | **?** | **?** | + | **Unclear** | Risk of reporting bias: Unclear if diagnostic criteria have been fulfilled in all RA cases. |
| Hartvigsen [68] | 2004 | **?** | +  + | **?** | **–** | **Unclear** | Uncertain if outcome definition applied to all cases. Risk of reporting and recall bias. |
| Hartvigsen [69] | 2006 | **?** | + | **?** | + | **Unclear** | Uncertain if outcome definition applied to all cases. Risk of reporting and recall bias. |
| Hartvigsen [70] | 2008 | + | + | **?** | **–** | **Unclear** | Uncertain if outcome definition applied to all cases. Risk of reporting and recall bias. Unclear if responders are representative of the target population. |
| Haugen [26] | 2011 | **?** | **?** | + | **?** | **Unclear** | Risk of sampling and non-response bias |
| Henry [45] | 2000 | **?** | **?** | + | + | **Unclear** | Unclear if responders are representative of the target population of women. |
| Hicks [71] | 2008 | **?** | **?** | **?** | **?** | **Unclear** | Unclear if responders are representative of the target population. |
| Hill [88] | 2010 | **?** | **?** | **?** | **?** | **Unclear** | Risk of sampling and non-response bias Unclear if responders are representative of the target population. |
| Holt [46] | 2002 | **?** | **?** | + | + | **Unclear** | Unclear if responders are representative of the target population |
| Jacobs [72] | 2006 | **?** | **?** | **?** | **–** | **Unclear** | Unclear if responders are representative of the target population. |
| Jinks [94] | 2008 | **?** | + | + | + | **Low** | Low risk of bias compared to target population. |
| Jordan [27] | 2007 | **?** | **?** | **?** | + | **Unclear** | Unclear if responders are representative of the target population. |
| Keenan [73] | 2006 | **?** | **?** | **?** | + | **Low** | Well described (via referenced article(s)) |
| Kenny [47] | 2009 | **?** | **?** | + | **–** | **High** | Non-random and small study sample. Unclear if responders are representative of the target population. MSK definition/criteria not stated. |
| Kim [28] | 2010 | **?** | **?** | **?** | + | **Unclear** | Unclear if responders are representative of the target population. |
| Kotz [54] | 2004 | **?** | **?** | **?** | **–** | **High** | High risk of reporting bias from the participants as the non-validated outcome definition is vaguely described and normally is a clinical diagnosis. |
| Kwok [29] | 2011 | **?** | **?** | **?** | + | **Unclear** | Unclear if responders are representative of the target population. |
| Laiho [15] | 2001 | **?** | **?** | + | + | **Unclear** | Unclear if responders are representative of the target population. |
| Lespessailles [55] | 2009 | **?** | **?** | **?** | **?** | **Unclear** | Unclear if responders are representative of the target population. |
| Mannoni [30] | 2003 | **?** | **?** | + | + | **Low** | Minimal non-response bias as the adjusted response rate is high. |
| Menz [96] | 2005 | **?** | **?** | + | **?** | **Low** | Low risk of selection bias as the non-response analysis showed no difference |
| Meyer [74] | 2007 | + | + | + | **?** | **Low** | Well described study when additional information on study sample from articles and website are obtained |
| Mickle [97] | 2010 | **?** | **?** | + | **?** | **High** | High risk of non-response bias |
| Miro [75] | 2007 | +  + | + | + | + | **Low** | Well described study. |
| Muraki [31] | 2009 | **?** | **?** | **?** | + | **Unclear** | Unclear if responders are representative of the target population. |
| Mølgaard [98] | 2010 | + | **?** | + | + | **Low** | Well described study. |
| Natvig [76] | 2004 | **?** | **?** | + | + | **Unclear** | Unclear if responders are representative of the target population. |
| Naves [48] | 2005 | **?** | **?** | **?** | + | **Unclear** | Unclear if responders are representative of the target population. |
| Neovius [16] | 2010 | + | + | + | + | **Low** | Includes the whole population through patient registries. Well described article. |
| Ollivier [17] | 2004 | **?** | **?** | + | + | **Low** | Low risk of non-response bias, due to the high response rate. |
| Parsons [77] | 2007 | **?** | **?** | + | + | **Unclear** | Risk of selection bias. |
| Peat [92] | 2006 | **?** | **?** | **?** | + | **Unclear** | Unclear if responders are representative of the target population. |
| Picavet [78] | 2003 | + | **?** | **?** | + | **Low** | Questionnaire not validated, but is similar to other validated questionnaires |
| Picavet [18] | 2003 | + | **?** | **?** | **?** | **High** | High risk of reporting bias from the participants as the non-validated outcome definition is vaguely described and normally is a clinical diagnosis. |
| Rasch [19] | 2003 | + | + | + | + | **Low** | NHANES III is well described elsewhere and shows a low risk of bias |
| Riise [20] | 2000 | + | + | + | + | **Low** | Low risk of reporting bias as it is the only rheumatology department and low risk of sampling bias, as it is the whole population in that region. |
| Saks [56] | 2001 | + | **?** | **?** | **?** | **Unclear** | No clear outcome definition. Unclear if the definition of the MSK disorder is validated. |
| Salaffi [79] | 2005 | + | **?** | **?** | + | **Unclear** | Unclear if responders are representative of the target population. Risk of recall bias. |
| Sanfélix-Genovés [49] | 2010 | + | **?** | + | + | **Unclear** | High risk of non-response bias. |
| Santos-Eggimann [80] | 2000 | + | **?** | + | + | **Low** | Well described study. |
| Shin [50] | 2010 | **?** | **?** | + | + | **Unclear** | Risk of selection bias. Unclear if study sample is representative of the target population. |
| Stranjalis [81] | 2004 | **?** | **?** | + | + | **High** | Risk of selection bias as stated by the authors. Unclear if study sample is representative of the target population. |
| Strine [82] | 2007 | **?** | **?** | **?** | + | **Unclear** | Risk of non-response bias. Unclear if sample is representative of the target population. |
| Sudo [32] | 2008 | **?** | **?** | **?** | + | **Unclear** | Unclear if responders are representative of the target population. |
| Suka [83] | 2009 | **?** | **?** | **?** | + | **High** | Risk of selection bias. Unclear if study sample is representative of the target population. |
| Symmons [21] | 2002 | **?** | **?** | + | + | **Unclear** | Risk of selection bias. Unclear if study sample is representative of the target population. |
| Thomas [87] | 2004 | **?** | **?** | + | + | **Unclear** | Unclear if responders are representative of the target population. |
| Vestergaard [52] | 2005 | + | + | + | + | **Low** | Low risk of bias: study uses national registries on the whole population. |
| Vogt [84] | 2003 | **?** | **?** | **?** | **?** | **Unclear** | Risk of recall bias. Unclear if study sample is representative of the target population. |
| Webb [85] | 2003 | **?** | **?** | **?** | + | **Unclear** | Small risk of selection bias. Unclear if study sample is representative of the target population. |
| Werner [57] | 2003 | **?** | **?** | **?** | **?** | **Unclear** | Risk of information bias. Unclear if study is representative of the target population. |
| Yang [51] | 2004 | **?** | **–** | + | + | **High** | High risk of selection bias. Unclear if study sample is representative of the target population (although this issue is discussed by the authors) |
| Yaron [86] | 2011 | **?** | **?** | + | + | **Unclear** | Unclear if study sample is representative of the target population (no description of non-responders). |
| Yoshida [34] | 2002 | + | **?** | + | + | **Unclear** | High risk of non-response bias. |
| Yoshimura [33] | 2009 | **?** | **?** | + | + | **Unclear** | Risk of selection bias: Ascertainment procedures are unclear. Unclear if study sample is representative of the target population. |
| Yoshimura [35] | 2009 | **?** | **?** | + | + | **Unclear** | Unclear if responders are representative of the target population. |
| Zhang [36] | 2002 | **?** | **?** | **?** | + | **Unclear** | Unclear if selection bias is present. Unclear if study sample is representative of the target population |
